# Supplementary material for: Stability and learning in excitatory synapses by nonlinear inhibitory plasticity
Source: PLoS Comput Biol. 2022 Dec 2;18(12):e1010682. doi: 10.1371/journal.pcbi.1010682 (PMC9718420; doi:10.1371/journal.pcbi.1010682)
Supplement: Attachment — Submitted filename: review.pdf [file pcbi.1010682.s005.pdf]

The authors propose a novel nonlinear inhibitory plasticity rule that stabilises runaway excitation which arises from a nonlinear Hebbian excitatory plasticity rule. They use an approach based on the activity of populations of neurons and describe such plasticity rules based on the average firing-rate of the neurons of each population. This neuron model allows for elegant mathematical analyses to be made, which are complemented by simulations. The authors also define a sliding threshold for LTD and LTP in both excitatory and inhibitory plasticity rules which avoids extra fine tuning of parameters. As a result of the combination of both rules together, the authors show that a postsynaptic neuron forms a tuning curve when receiving independent inputs, which is diverse in a recurrent network, i.e., each neuron of the network responds preferentially to a distinct input feature after learning. The stability of synaptic dynamics is at the core of any type of learning and unveiling the nature of stabilising mechanisms are essential for us to understand how the brain learns (e.g., form memories). The authors propose an interesting new model of inhibitory plasticity which, differently than previous models, can stabilise excitatory plasticity while allowing for learning to still happen at excitatory synapses. Moreover, I like the analytical approach used in the manuscript which allow for conclusions to be drawn without extensive simulations and parameter sweeps.

As interesting as these results are, however, I have major concerns about the relevance of the results when related to previous models of synaptic plasticity. Previous work, including the work by the authors have solved the so-called "temporal paradox" (as described by the authors in the Discussion section) with LTP/LTD sliding threshold (Bienenstock et al., 1982; Gjorgjieva et al., 2011; Zenke et al., 2013; Wu et al., 2020), normalization of weights (Litwin-Kumar and Doiron, 2014; Wu et al., 2020), and heterosynaptic plasticity (Zenke et al., 2015; Wu et al., 2020; Field et al., 2020; Kirchner and Gjorgjieva, 2021). I expand on this and other points below which I separated in major and minor points.

Major points

1. *Comparison to previous models of excitatory plasticity.* The excitatory synaptic plasticity model used by the authors (Eq. 1) resembles the one proposed by Leon Cooper in the 70s (Cooper, 1973; Cooper et al., 1979), which was modified later by Bienenstock, Cooper, and Munro (Bienenstock et al., 1982) to include a sliding-threshold for LTD/LTP. The sliding threshold does not only allow for selectivity to emerge, but also stabilises postsynaptic activity as shown more recently in spike-based versions of the BCM model (Gjorgjieva et al., 2011; Zenke et al., 2013). How would the BCM model (with sliding threshold) behave in the simulation paradigm used by the authors? Would an excitatory plasticity model with sliding threshold together with a linear inhibitory plasticity model be unstable? I would like to suggest repeating the simulations (and analysis) done in Fig. 1 for a model with the sliding threshold as a comparison between the well established BCM model and the one used by the authors (maybe as a supplementary figure).

   Similar plasticity rules have been used by Litwin-Kumar and Doiron (2014) to study the formation of assemblies – excitatory learning rule similar to Eq. 1 and linear inhibitory plasticity model similar to Eq. 2. A comparison would be interesting (maybe in the discussion section).

   Additionally, how does this model relate to the previous model from Kirchner and Gjorgjieva (2021) and Field et al. (2020)?

2. *Robustness to noise and time-varying inputs.* The synaptic plasticity model used by the authors to describe changes in excitatory synapses has two fixed-points ($\dot{w}^{EE} = 0$) for the excitatory activity: $\nu^E = 0$ and $\nu^E = c^E_{post}$ according to Eq. 1. The first fixed-point ($\nu^E = 0$) is stable, and the second ($\nu^E = c^E_{post}$) is unstable as explained by the authors. The nonlinear inhibitory synaptic plasticity model proposed by the authors achieve stability by setting the postsynaptic firing-rate at $\nu^E = c^E_{post}$, i.e., the **unstable** fixed-point for excitatory plasticity. As long as $\nu^E = c^E_{post}$ with *machine precision* (in the simulations), the system (postsynaptic neuron's activity) will remain constant. Would dynamical inputs (that change over time), or noise, result in excitatory weights drifting away from the unstable fixed-point, even though the nonlinear inhibitory plasticity mechanism may be constantly keeping $\nu^E \approx c^E_{post}$? It is not clear whether the system is stable when the input is constantly changing over time and/or noise is added to system. To test stability of this model, I would thus like to suggest two additional simulations. First, to add noise to the term $\nu^E(t)$, repeating the simulation from Fig. 2C for longer periods of time, i.e., minutes or hours. Second, to use an input signal ρ^E that changes over time with, e.g, a sinusoidal signal.

3. *Unstable initial conditions.* Figs. 1E and 2B: the linear inhibitory plasticity model has a set of initial conditions (for w^{EE} and w^{EI}) that results in runaway excitation, while the nonlinear inhibitory plasticity model has a set of initial conditions that results in a silent postsynaptic neuron. Even though runaway excitation is

presumably "worse" than no postsynaptic activity, the nonlinear inhibitory learning rule still fails to bring the postsynaptic activity to a steady non-zero firing-rate for a large set of initial conditions. This seems to me like a "weakness" of the nonlinear inhibitory plasticity model which is not mentioned in the text, apart from lines 672/673 in the Methods. Would it be beneficial to add a linear term to compensate for this? In this case the model would still be nonlinear but without any "forbidden" regions on the w^{EE} w^{EI} plane.

4. *Population versus individual neuron's dynamics.* The authors use, for most of the analysis/simulations, a single population of presynaptic excitatory neurons, similarly for postsynaptic neurons and presynaptic inhibitory neurons (Figs. 1 to 5, and S1 Fig); $N^E = N^I = 1$. When both N^E and N^I are greater than 1, how does the activity of the different populations evolve over time? Fig. 6E shows the evolution of weights, but it's unclear how the different populations' activities evolve. Additionally, the authors claim that because the set-points c^E_{post} and c^I_{post} reach a steady state for single populations ($N^E = N^I = 1$), it is safe to assume that they will have constant values. However, it's not clear to me what happens when both N^E and N^I are greater than one if the set-points are also evolving over time. I would like to suggest confirming this claim by running simulations, such as the ones described in Fig. 6, with both set-points evolving over time.

5. *Neuron model.* Many of the results, and consequently, conclusions of the manuscript rely on the choice of the neuron model (Eqs. 7 and 8). Using a linear rectifier as a model to describe the firing-rate of the neurons is a valid choice, especially for mathematical tractability. Nevertheless, most of the results depend on this choice. For example, the dependence of excitatory plasticity on the presynaptic rate and inhibitory-to-excitatory weights (shown, e.g., in Figs. 1C and 4E) is indirect: excitatory plasticity depends only on ρ^E and ν^E directly (Eq. 1). For example, in Fig. 1C the different curves assigned to different w^{EI} reflect changes caused by distinct ν^E that are a consequence of changing w^{EI} while keeping the other parameters (ρ^E, ρ^I, N^E, N^I, and w^{IE}) fixed. To clarify the effect of these parameters I would like to suggest including Eqs. 7 and 8 in the main text so that it's easier for a reader to understand how changes in, e.g., w^{EI}, affect the curves in Fig. 1C. Additionally, it would be interesting to have a 3D color plot (such as the one in Fig. 6E) showing the changes in weight ($\dot{w}^{EE}$, color) as a function of ρ^E (e.g., x-axis) and ν^E (e.g., y-axis).

6. *Assuming static c^E_{post} and c^I_{post} (from page 9 onward).* As mentioned above (point 4), the assumption that the dynamics given by Eq. 20 will lead to constant c^E_{post} and c^I_{post} for the case involving multiple populations might not hold true. I would like to suggest carefully checking if that assumption holds, especially in case noise or varying inputs are used. Additionally, the dynamics proposed for c^E_{post} and c^I_{post} seem to create a plasticity mechanism that is non-local (Eq. 20). Could the authors clarify why they chose to not use the classic BCM sliding threshold?

7. *Fixed E/I ratio.* The authors define E/I ratio as $R^{E/I} = w^{EE}/w^{EI}$ and show that this quantity is regulated by the combination of excitatory and inhibitory nonlinear plasticity rules (Eqs. 1 and 3). They calculate this quantity (Eq. 6) and state that "The nonlinear inhibitory plasticity rule establishes a fixed excitatory and inhibitory weight ratio". In what sense is it fixed? I would consider as "fixed" if the value is independent of, e.g., inputs, and thus being a constant. The fact that it depends on the level of excitatory and inhibitory inputs means that it's not fixed; is that correct? Additionally, the typical definition of E/I *balance* is related to input currents. In this neuron model this would be $E/I = (w^{EE}\rho^E)/(w^{EI}\nu^I)$. What happens with this quantity?

   The authors claim that the combination of the nonlinear excitatory and inhibitory plasticity model (Eqs. 1 and 3) create an E/I set-point. However, as shown in Figs. 2D and 5A, the E/I ratio is defined by a line attractor instead of a single *set-point*. I would like to suggest clarifying this in the text.

Minor points

8. Abstract: "Synaptic changes underlie learning and memory formation in the brain." I suggest adding the word "hypthesised" here.

9. Abstract: "But synaptic plasticity of excitatory synapses on its own is unstable, ..." This is sentence is inaccurate if synaptic plasticity of excitatory synapses is defined as any mechanism that is involved in modifications of excitatory synapses, e.g., synaptic scaling (Wu et al., 2020). Are the authors referring here to Hebbian plasticity? I suggest rephrasing this sentence.

10. Abstract: "... leading to unlimited growth of synaptic strengths without additional homeostatic mechanisms." Following the point above (point 9) – is that related to Hebbian plasticity of excitatory synapses? In this case I would like to point-out to an additional undesirable state, which is silence, i.e., zero firing-rate. In this case Hebbian plasticity of excitatory synapses can lead to either runaway excitation or complete silence. I suggest rephrasing and clarifying this sentence.

11. Abstract: "We identify two key features of inhibitory plasticity, dominance of inhibition over excitation and a nonlinear dependence on the firing rate of postsynaptic excitatory neurons whereby inhibitory synaptic strengths change in the same direction as excitatory synaptic strengths." It seems that the *nonlinear dependence on the firing-rate of postsynaptic excitatory neurons* is explicit from the model, and it is thus not a key feature **identified** by the authors, but a feature **imposed** by the authors. I would like to suggest rephrasing this sentence.

    The last part of the sentence is confusing to me ("whereby inhibitory synaptic strengths change in the same direction as excitatory synaptic strengths.") Is this related to *dominance of inhibition over excitation* **or** *nonlinear dependence on the firing rate of postsynaptic excitatory neurons*?

12. Abstract: "... by this novel inhibitory plasticity achieve ..." I would like to suggest adding the word *model* after *plasticity*.

13. Abstract: "... achieve a fixed excitatory/inhibitory set-point in agreement with experimental results." The excitatory/inhibitory set-point referred here is for weights and not currents as shown experimentally. I would like to suggest rephrasing this sentence to accurately describing the results from the model.

14. Line 2: "Learning and memory formation in the brain are implemented by synaptic changes" I would like to suggest adding the word *hypothesis* or *theorised*, as references 1 and 2 are not confirmation of this claim but hypotheses/theories.

15. Line 77: "Here, a population of presynaptic excitatory neurons projects to a population of inhibitory neurons and both populations project to a postsynaptic excitatory neuron." The mean-field type of modelling (e.g., Eqs. 1, 2, and 3) typically assumes average activity of a population being described by the variables. Could the authors clarify why ν^E describes a single neuron and not a population?

16. Eq. 1: Are the units correct? From Eq. 7 it looks like w^{EE} is unit-free, thus the left side of Eq. 1 is unit-free given that the unit of τ_w^E is milliseconds (representing time). The variables ρ^E, ν^E, and c_{post}^E have the same unit, Hz, so the right side of Eq. 1 has the unit of Hz^3. Wouldn't it be better to use a learning rate with a *correcting* unit instead of a characteristic time?

17. Line 100: "In our framework, inhibitory neurons can affect excitatory plasticity in three equivalent ways." These three ways reflect the author's choice of neuron model (Eqs. 7 and 8). I would like to suggest including these two equations in the main text to explicitly show how the neuron model affects the plasticity rule. This way it's easy to follow that, e.g., $\nu^E = \rho^E w^{EE} - (\rho^E w^{IE} + \rho^I)w^{EI}$ for $N^E = N^I = 1$ (from the steady states of Eq. 7 and 8).

18. Eq. 4: The authors derived this equation considering the steady-state of ν^E. Could the authors do the same for ν^I to write the left side of the inequality as $N^I(N^E\rho^E w^{IE} + \rho^I)^2/\tau_w^I$?

19. Eq. 5: Similar to the point above (point 18). Could the authors rewrite the right side of the equation using the steady state of ν^I?

20. Line 202/203: "When the excitatory postsynaptic threshold is lower than the inhibitory postsynaptic threshold ($c_{post}^E < c_{post}^I$) ..." I would like to suggest adding here what happens in the other case, i.e., when $c_{post}^E > c_{post}^I$.

21. Line 210/211: "Motivated by experimental findings and theoretical considerations [44], we proposed that these thresholds can be dynamically regulated in opposite directions (Fig 3D; see Methods). When the postsynaptic rate is lower than the excitatory postsynaptic LTD/LTP threshold ($\nu^E > c_{post}^E$), the excitatory postsynaptic LTD/LTP threshold should decrease, while when the postsynaptic rate is higher than the threshold ($\nu^E < c_{post}^E$), the excitatory threshold should increase." For the best of my knowledge, the description of the threshold dynamics for excitatory plasticity is exactly how the BCM theory was first described (Bienenstock et al., 1982). I would like to suggest rephrasing this first sentence as the authors are not proposing a new form of sliding threshold, but proposing that inhibitory plasticity also has one.

22. Line 219: "... these dynamic ..." The letter **s** is missing: dynamics.

23. Line 224: "The generation of such heterogeneous postsynaptic rates is consistent with experimental observations in multiple brain regions [45]." It looks like the postsynaptic firing-rate is either $\nu^E \approx 0.95$ Hz or $\nu^E \approx 0.99$ Hz in Fig. 3F. The statement that this constitutes a heterogeneous postsynaptic rate that is consistent with experimental findings is a bit too strong. I would like to suggest adding more simulations (or mathematical analysis) to show heterogeneous firing-rate in, e.g., a histogram plot of firing-rates or to change this sentence.

24. Line 228/229: "Since even if they are unequal, excitatory and inhibitory LTD/LTP thresholds can be dynamically matched, from now on we assumed that they are equal and static." Please see point 6.

25. Line 242-246: "For example, it has been shown that input perturbations ..." I would like to suggest moving these sentences to the Discussion section as it does not present new results but discuss them in the scope of previous work.

26. Line 247-250: "Our framework can even predict the steady values ..." This is only true for the specific choice of neuron model. I would like to suggest rephrasing this sentence so that it's clear that it depends on the neuron model (see point 5).

27. Line 256/257: "Such a shift in the plasticity threshold for excitatory synapses has been measured ..." The threshold that changes in the model is for the presynaptic activity (Fig. 4E). Is it the same for the experimental results cited here?

28. Eq. 6: This equation is valid for the neuron model used here (see point 5). I would like to suggest adding this to the text. Additionally (see point 7), the excitatory-to-inhibitory ratio (E/I) is usually calculated based on input currents, which would be $R^{E/I} = \frac{\rho^E w^{EE}}{\nu^I w^{EI}}$ with the neuron model used by the authors (Eq. 7).

29. Eq. 6: To arrive in this equations the system is in a steady state, and thus $\nu^I = N^E w^{IE} \rho^E + \rho^I$, which results in $R^{E/I} = \frac{N^I(N^E \rho^E w^{IE} + \rho^I) w^{EI} + c_{post}}{N^E \rho^E w^{EI}}$. I would like to suggest explicitly showing the calculation considering the parameters of the neuron model.

30. Line 273: "For strong I-to-E weights w^{EI}, the E/I ratio approximates to $R_\infty^{E/I}$ ..." This condition is valid for $N^I \nu^I w^{EI} \gg c_{post}$ according to Eq. 6. Given that the steady state of ν^I can be calculated analytically, this expression becomes $N^I(N^E \rho^E w^{IE} + \rho^I) w^{EI} \gg c_{post}$. I would like to suggest clarifying the contribution of the parameters of the model, especially ρ^E, N^E, w^{IE}, and ρ^I.

31. Line 273: "... E/I ratio approximates to $R_\infty^{E/I} = \frac{N^I \nu^I}{N^E \rho^E}$ ..." Considering the steady state for ν^I, this term becomes $R_\infty^{E/I} = \frac{N^I(N^E \rho^E w^{IE} + \rho^I)}{N^E \rho^E}$. I would like to suggest adding this extra step to clarify how the neuron model's parameters influence the ratio E/I.

32. Line 278/279: "... which needs to be counteracted by even more inhibitory LTP to stabilize the weights." Wouldn't it be *to stabilize the weight dynamics*?

33. Line 284/285: "matching experiments [26]." Which experiments do the authors refer to?

34. Line 311/312: "we applied a disinhibitory signal by decreasing the external excitatory input onto the inhibitory populations." I could not find an equation related to this implementation in the Methods. Considering Eqs. 7 and 8, changes in the excitatory input, ρ^E, affect both ν^E and ν^I. I would like to suggest adding a variable to Eq. 8 to indicate how disinhibition is implemented, and the parameter used for this variable in Fig. 6.

35. Lines 351 to 354: Aren't these "two key features" strictly necessary for the stability?

36. Line 355: "... with a suitable mechanism that enables self-adjusting of the plasticity thresholds ..." Doesn't this mechanism make the plasticity rules non-local? It would be worth mentioning this in the discussion.

37. Line 360: ".. in agreement with experiments [26]." Which experiments? Could the authors elaborate on this?

38. Lines 372 to 374: "However, the spatial and temporal scales for integrating Hebbian and homeostatic plasticity have remained an open question [17,20,21]." Isn't heterosynaptic plasticity (Zenke et al., 2015; Field et al., 2020; Kirchner and Gjorgjieva, 2021) a solution for this question?

39. Line 424 to 426: "More dominant means that I-to-E weights need to change with a higher magnitude at each time step compared to E-to-E weights, for all postsynaptic rates." Isn't this finding contradictory to the experimental results from Froemke et al. (2007); particularly Fig. 4 from this experimental paper?

40. Lines 450 to 453: "This is consistent with several experimental studies which have suggested that inhibitory plasticity keeps an E/I ratio set-point [9,26,32,36,62,80–83]." Isn't the set-point for both excitatory and inhibitory rules defined by the postsynaptic firing-rate, ν^E (Eqs. 1 and 3)? Additionally, from Eq. 6, the E/I ratio is described as a result from other variables, such as ρ^E, and thus changes according to the presynaptic firing-rate – in this case it would not be a *set-point*, but a by-product of the firing-rate set-point.

41. Line 461/462: "The emergence of an E/I ratio set-point and the stabilization of rates driven by the novel inhibitory plasticity rule ensure a fixed E/I balance" See points above.

42. Line 552 to 554: "Functionally, our proposed inhibitory plasticity can establish and maintain a fixed E/I ratio set-point. At this set-point, no synaptic plasticity is induced, i.e. plasticity is "off"." According to Eqs. 1 and 3, plasticity is off when $\nu^E = c^E_{post} = c^I_{post}$, which happens for specific combinations w^{EE} and w^{EI}. I would like to suggest rephrasing these sentences to clarify that it's not the E/I ratio set-point that *switches* plasticity off, but the firing-rate set-point.

43. Eqs. 12 to 19: I would like to suggest using the steady state value of ν^I here so that's easier to know how the neuron model's parameters affect this quantity.

44. Line 634/635 and Eqs. 18 and 19: "The line which separates stable from unstable initial weights can be calculated by taking the ratio of Eq 9 and Eq 10 and equating that to the slope of the line attractor (Eq 5)" Isn't Eq. 5 calculated for the nonlinear inhibitory plasticity model? From Fig. 1D: "Black cross marks crossover of the plasticity curves at which weight dynamics become unstable." Isn't the point separating stable from unstable initial weights given by $\dot{w}^{EE} = \dot{w}^{EI}$ for the linear inhibitory plasticity model?

45. Eq. 20: I could not find the definition of Δw^{EE} or Δw^{EI}. Is it $\dot{w}^{EE}$ and $\dot{w}^{EI}$? If that's the case, doesn't it make the learning rules non-local? Or would it be that each synapse has a different c_{post}? I would expect that a sliding threshold defined by the BCM rule, such as in Gjorgjieva et al. (2011), would have similar results, and in this case the learning rule would remain local.

46. Lines 656 to 664: I find this paragraph a bit hard to follow. First, what results from ref. 26 are the authors linking to their model? Second, it's also not clear to me why using only 100 ms to calculate the points for Fig. 5. Third, without any noise all the points can be analytically calculated. Is there a reason not to show the analytical points?

47. Line 668/669: "We define a pattern to mean an increase in the input rate to 4 Hz for 100 ms of four neurons." This sentence is a bit confusing to me. What does *mean* refer to? Also, how would this be reflected in Eqs. 7 and 8?

48. Lines 669 to 671: Why 60 seconds? How sensitive are the results to this choice?

49. Line 672/673: "... specific inhibitory population as slow and gradual over a time course of 100 s to avoid complete silencing of the postsynaptic excitatory neurons." Would this happen with the linear inhibitory plasticity model as silent postsynaptic activity elicits a decrease in inhibitory weights when inhibitory neurons are active? See point 3.

50. Figures: I would like to suggest deleting "in" from "in [Hz]" – isn't it redundant to have "in" and brackets?

51. Figure 4: I could not find the definition of ρ^E_{base} and ρ^E_{distr} in the Methods. Would it be possible to add their definitions in the Methods section?

52. Figure 6 F: It is not clear why the neurons are organised in such a way in this plot. Does the distance between two coupled neurons reflect something? I would like to suggest clarifying this in the caption (or Methods) or changing the representation.

References

Bienenstock, E. L., Cooper, L. N., and Munro, P. W. (1982). Theory for the development of neuron selectivity: orientation specificity and binocular interaction in visual cortex. *Journal of Neuroscience*, 2(1):32–48.

Cooper, L. N. (1973). A possible organization of animal memory and learning. In Lundqvist, B. and Lundqvist, S., editors, *Collective properties of physical systems: Proceedings of the Twenty-Fourth Nobel Symposium*, page 252–264.

Cooper, L. N., Liberman, F., and Oja, E. (1979). A theory for the acquisition and loss of neuron specificity in visual cortex. *Biological Cybernetics*, 33(1):9–28.

Field, R. E., D'amour, J. A., Tremblay, R., Miehl, C., Rudy, B., Gjorgjieva, J., and Froemke, R. C. (2020). Heterosynaptic plasticity determines the set point for cortical excitatory-inhibitory balance. *Neuron*, 106(5):842–854.

Froemke, R. C., Merzenich, M. M., and Schreiner, C. E. (2007). A synaptic memory trace for cortical receptive field plasticity. *Nature*, 450(7168):425–429.

Gjorgjieva, J., Clopath, C., Audet, J., and Pfister, J.-P. (2011). A triplet spike-timing–dependent plasticity model generalizes the bienenstock–cooper–munro rule to higher-order spatiotemporal correlations. *Proceedings of the National Academy of Sciences*, 108(48):19383–19388.

Kirchner, J. H. and Gjorgjieva, J. (2021). Emergence of local and global synaptic organization on cortical dendrites. *Nature Communications*, 12:4005.

Litwin-Kumar, A. and Doiron, B. (2014). Formation and maintenance of neuronal assemblies through synaptic plasticity. *Nature Communications*, 5:5319.

Wu, Y. K., Hengen, K. B., Turrigiano, G. G., and Gjorgjieva, J. (2020). Homeostatic mechanisms regulate distinct aspects of cortical circuit dynamics. *Proceedings of the National Academy of Sciences*, 117(39):24514–24525.

Zenke, F., Agnes, E. J., and Gerstner, W. (2015). Diverse synaptic plasticity mechanisms orchestrated to form and retrieve memories in spiking neural networks. *Nature Communications*, 6:6922.

Zenke, F., Hennequin, G., and Gerstner, W. (2013). Synaptic plasticity in neural networks needs homeostasis with a fast rate detector. *PLoS Computational Biology*, 9(11):e1003330.
