# Supplementary material for: Stability and learning in excitatory synapses by nonlinear inhibitory plasticity
Source: PLoS Comput Biol. 2022 Dec 2;18(12):e1010682. doi: 10.1371/journal.pcbi.1010682 (PMC9718420; doi:10.1371/journal.pcbi.1010682)
Supplement: Attachment — Submitted filename: main.pdf [file pcbi.1010682.s006.pdf]

We thank all three reviewers for their insightful and constructive feedback. We have addressed all points raised by the reviewers in the revised manuscript by additional analysis and rewriting of the problematic text, adding one new main figure (Fig 6) and two new supplementary figures (S1 Fig, S3 Fig), and feel that the manuscript has greatly improved through these additional changes. To help with the next review, we have highlighted all changes in the manuscript in blue. In the following, we individually answer every comment of the reviewers and link them to the respective section in the manuscript:

Reviewer #1:

Summary

This is an interesting paper addressing an important topic of stability of operation of neuronal microcircuits in face of on-going synaptic plasticity. The authors propose a novel mechanism that may help to stabilize activity in a model of canonical feedforward excitatory/inhibitory microcircuit. The authors demonstrate that the proposed mechanism can also regulate, by affecting the rate of postsynaptic neuron, plasticity at excitatory synapses and thus serve as a mechanism of metaplasticity.

The paper will make valuable contribution towards mechanistic understanding of plasticity in neuronal microcircuits.

There are several points, however, that have to be addressed.

Comment 1

In the Abstract (may be also in the title), it should be clearly indicated that this is a purely modelling study.

Answer: We added the terms "model" and "modeling" in the Abstract.

Comment 2

Introduction L10-18: experimentally supported homeostatic mechanisms: synaptic scaling, heterosynaptic plasticity, plasticity of intrinsic excitability

... Part of the challenge is that the experimentally measured timescales of homeostatic mechanisms are too slow to stabilize the Hebbian runaway dynamics in computational models, sometimes referred to as the 'temporal paradox' of homeostasis [19–21].

similarly in the Discussion (lines 372-374).

The problem of fundamentally different timescales (seconds vs many hours and days) is true ONLY for synaptic scaling, which thus fundamentally cannot counteract positive feedback of Hebbian-type plasticity but nevertheless referred too most extensively. Heterosynaptic plasticity is induced on exact same time scale as Hebbian-type plasticity and intrinsic plasticity – on a similar time scale. So the 'temporal paradox' applies only to synaptic scaling, but not to heterosynaptic plasticity or intrinsic plasticity. Please explain this.

Answer: The reviewer is correct, the timescale problem is only related to synaptic scaling but not to heterosynaptic plasticity or intrinsic plasticity. To clarify that the temporal paradox is only related to synaptic scaling, we modified line 34-39 in the Introduction:

> Part of the challenge is that the experimentally measured timescales of *synaptic scaling* are too slow to stabilize the Hebbian runaway dynamics in computational models, *where much faster normalization schemes are used instead [26, 44, 30, 42, 34].* This is sometimes referred to as the 'temporal paradox' of homeostasis [47, 46, 45]. A related problem to the integration of plasticity and homeostasis is the trade-off between stability and flexibility. While stimulus representations need to be stable, for instance to allow long-term memory storage, the system also needs to be flexible to allow re-learning of the same, or learning of new representations [12]. *This has been successfully achieved in some circumstances. For example, implementing metaplasticity in the excitatory connections through*

a sliding threshold between potentiation and depression can generate weight selectivity and firing rate stability [4, 14, 47, 42]. Additionally, heterosynaptic plasticity has been modeled to stabilize synaptic weight dynamics, while still allowing learning [7, 39, 10, 20], including behavioral learning [6]. A strong candidate for stabilizing synaptic weights is the induction of homosynaptic LTP (LTD) together with heterosynaptic LTD (LTP) at nearby synapses, referred to as the 'Mexican hat' profile of homo- and heterosynaptic plasticity [41, 33].

We also now write in the discussion in lines 434-445:

> *At the same time, computational studies have included multiple homeostatic mechanisms, some of them the same as the experimental ones, to stabilize rates and weight dynamics, including upper bounds on the E-to-E weights, normalization mechanisms [31, 29, 26, 44, 42, 34], metaplastic changes of the plasticity function [4, 14, 47, 43, 42], heterosynaptic plasticity [39, 10, 20] and intrinsic plasticity and synaptic scaling [42].* However, the spatial and temporal scales for integrating Hebbian and homeostatic plasticity have remained an open question [37, 46, 45]. *This is especially the case for synaptic scaling which experimentally operates on timescales too slow to counteract the faster Hebbian synaptic plasticity (hours and days, vs. seconds and minutes). Heterosynaptic plasticity has been suggested as a more natural solution to the 'temporal paradox' problem since it operates on a similar timescale as Hebbian plasticity [7, 39, 10].*

Comment 3

Introduction

... However, how exactly synaptic plasticity and homeostatic mechanisms interact to control synaptic strengths, and yet enable learning, is still unresolved [16–18].

While indeed there is no consensus on this issue, there are at least attempts to do so. The ability of experimentally-observed heterosynaptic plasticity to counteract destabilizing effects of STDP on synaptic weights and activity, while allowing learning was demonstrated in model neurons and simple networks (Chen et al 2013; Volgushev et al 2016). In fact, even evidence for the relevance of heterosynaptic plasticity for behavior learning was presented (Chasse et al 2020). Also 'Mexican hat' profile of homo- and hetero-synaptic plasticity can provide a mechanism for balancing synaptic changes (White et al 1990; Royer, Pare 2003).

Answer: We agree with the reviewer that several studies have shown how heterosynaptic plasticity can have stabilizing effects. We added this information into the Introduction in line 34-39 and Discussion in line 434-445 (see our answer to Comment 2).

Comment 4

Excitatory plasticity rule used in this study is highly unbalanced, with only weak depression and strongly dominating potentiation. Also rate for LTD/LTP threshold is low (1 Hz). However in physiological experiments low frequency stimulation with 1,3 or 5 Hz rather induces LTD, and LTP is induced at 10-20 Hz or higher. So frequency threshold in conventional BCM rule would be around 10 Hz. I see that there is no direct translation of rates from rate models to conventional BCM/STDP/tetanus induction protocols, and 1 Hz threshold might be 'typical' for rate models, but it would helpful to discuss this issue.

Answer: We thank the reviewer for this comment. We note that the choice of the LTD/LTP threshold parameter in the model is arbitrary. Choosing a different threshold e.g. 10 Hz instead of 1 will not affect any of our results, including the main finding that the excitatory and inhibitory LTD/LTP thresholds should overlap. We agree that mentioning the different firing rates for plasticity induction in experiments is relevant, so we added a brief discussion in the Methods section at line 692-696:

> *During experimental induction of plasticity, low frequency stimulation (1,3 or 5 Hz) induces LTD, while high frequency stimulation (10-20 Hz) induces LTP [21]. Therefore, a natural value of the*

LTD/LTP threshold is between 5 and 10 Hz. We chose 1 Hz as the LTD/LTP threshold (Table 1), nonetheless, our findings will still hold with higher LTD/LTP thresholds.

Comment 5

Throughout the results and figures, please always indicate postsynaptic firing rates since it is critical for understanding in which region of plasticity curve the model is operating. Yes, firing rate is indicated in most cases, please add it where it's missing.

Answer: We have followed the reviewer's suggestion and ensured that the postsynaptic firing rate is always clearly indicated. The three main changes were to Figures 1, 5 and 7. Specially, we expanded the caption of Fig 1D to denote the postsynaptic rates for which the system with linear inhibitory plasticity becomes unstable:

> The black cross marks *the postsynaptic rate where the plasticity curves cross* beyond which the weight dynamics become unstable.

In Fig 5, we noted that the firing rates are similar as in Fig 2. In Fig 7E, we added the trace of the postsynaptic firing rate, see also our answer to Comment 10.

Comment 6

Fig 4 – add a plot of plasticity rules used in this simulation.

Answer: We now state which plasticity rule was used in the caption of Fig 4 and in line 255-257:

> Excitatory and inhibitory LTD/LTP thresholds can be dynamically matched *under most conditions, even if they are unequal (S3 Fig). Therefore,* from now on we assumed that they are equal and static *(as shown in Fig 2A).*

Comment 7

... their potentiation (Fig 4C). The response to these perturbations is consistent with previous experimental results. For example, it has been shown that input perturbations via sensory deprivation decrease inhibitory activity [49–51].

Deprivation effects are developed on timescale of hours/days; in the model (Fig. 4C) within less than a second. I would say you are introducing here an 'inverse temporal paradox' trying to say that a process completed within a second could underlie day-long gradual changes. Please explain/discuss.

Answer: Thanks for this relevant comment. The reviewer is indeed correct in that the timescales of sensory deprivation-induced plasticity are much longer (hours to days) than the plasticity timescales in the model (seconds to minutes). We have now moved the relevant sentences to the Discussion section at lines 507-516 (based on Rev 2, comment 25) where we acknowledge this mismatch of timescales, and further write:

> *We note that the plasticity induced by these sensory deprivation experiments occurs on much longer timescales of hours to days (see e.g. [13, 18]) compared to the shorter plasticity timescales of seconds or minutes in our model, suggesting that other mechanisms than the proposed nonlinear inhibitory plasticity drive the experimentally observed changes. Moreover, in our model we instantaneously and permanently change the input firing rate in contrast to the more complex changes in input patterns occurring during sensory deprivation. Therefore, the applied perturbation in our model could be better related to direct simulation of input pathways when similarly fast LTD/LTP threshold changes have been measured experimentally [16, 3, 1]).*

In other words, the sensory deprivation experiments are just one example of a perturbation in terms of the applied excitatory input. Perhaps more appropriate examples would be direct or acute perturbations where similarly fast changes (for e.g. of the plasticity thresholds) as in our model might be observed.

Comment 8

Same problem as above, very different time scales, holds for prediction of the steady state of E/I ratio (from line 269). In the model, steady state is reached within few seconds; this could be time scale of sensory adaptation, but not of say deprivation-induced changes. Please explain/discuss.

Answer: Unlike the previous comment which is about Figure 4 (where perturbing the excitatory input is related to sensory deprivation), we believe that this comment is about Figure 5 (maintenance of a fixed E/I weight ratio). In the context of Figure 5 we refer to experiments where inhibitory plasticity was induced in the auditory cortex [9]. The timescales of this experiment are much faster than sensory deprivation, i.e. 5-10 minutes for the induction of plasticity after which new E/I ratios are observed. Therefore, here the timescale comparison in the experiments and the model is appropriate. We have added to the Discussion in line 595:

> *In these experiments, a change in E/I ratio is observed on the timescale of induction of plasticity (5̃-10 min) [9].*

Comment 9

... Decreasing the excitatory input rate decreases the excitatory presynaptic LTD/LTP threshold, hence

This sounds counter intuitive – decreasing presynaptic rate might make it more difficult to reach fixed postsynaptic threshold rate. Or, because of inhibitory-dominated circuit decrease of presynaptic rate would have strong disinhibiting effect which 'overrules' the decrease of presynaptic input? Or am I missing something? Please explain. Also, in Fig 6B it's the other way round –presynaptic LTD/LTP threshold increases for lower presynaptic rate, and decreases for higher. This is more intuitive. Please explain the difference between Fig 4E and 6B.

Answer: The discrepancy between Fig 4E and Fig 7B (Fig 6B in the previous submission of our manuscript) was due to a wrong color scheme that we used in the legend of Fig 7B. The color scheme has now been corrected, and Fig 7B is now consistent with Fig 4E.

To address why decreasing presynaptic rates counterintuitively leads to lower presynaptic LTD/LTP thresholds, we dissect the inhibitory plasticity rule. A permanent decrease in the presynaptic firing rate leads to a transient decrease in the postsynaptic firing rate (Fig 4B, blue dashed line) which, as a consequence, leads to LTD at the excitatory and the inhibitory weight (Fig 4C, blue and red dashed lines). Since the "goal" of inhibitory plasticity is to bring the postsynaptic firing rate to c_{post}, the decrease of inhibitory synapses ends up stronger than the decrease of excitatory synapses (reflected in Fig 4C). Therefore, even for a smaller presynaptic firing rate, the postsynaptic neuron will eventually reach a firing rate of c_{post} in its stable configuration. Hence, when plotting the excitatory weight change as a function of the presynaptic excitatory rate (Fig 4E), the LTD/LTP threshold will fall exactly at the point of the presynaptic excitatory rate where the system is stable and no further plasticity is be induced (in the absence of further perturbations). To make this point, we added the following sentence in lines 279-282:

> *The reduction in the LTD/LTP threshold follows from the relatively stronger depression of inhibitory compared to excitatory weights allowing the excitatory postsynaptic neuron to fire at the target rate even when the excitatory input is decreased.*

Comment 10

... We found that disinhibiting the unspecific inhibitory population does not selectively potentiate E-to-E weights, and hence does not generate competition among the different inputs. In contrast, disinhibiting all ten

specific inhibitory populations strongly increases the E-to-E weights corresponding to only a subset of inputs, a process also called receptive field formation (Fig 6E).

Please add postsynaptic firing rate trace to Fig 6E.

In the first case, please explain why changes of the postsynaptic firing rate did not induce plasticity. Were changes of firing rate after nonspecific disinhibition same as after the specific?

In the second case, inputs were probably disinhibited non-equally? Please provide details. And, if disinhibition was same for all inputs, please explain why changes of weights were heterogeneous (or random?).

Answer: We have now added the trace of the postsynaptic firing rate to Fig 7E. As one can see, disinhibiting via the unspecific inhibitory population only minimally disrupts firing rates, therefore, almost no plasticity is induced. In contrast, disinhibiting via the specific inhibitory populations leads to higher firing rate fluctuations. After the postsynaptic neuron has learned its receptive field, the postsynaptic firing rate increases above zero only when a single input, namely input number 3, is presented, due to the potentiated excitatory weight.

Related to the second part of the question, all inputs were inhibited equally. The heterogeneity in weight changes was due to the random order of the presentation of the inputs, meaning that by chance some inputs are more often presented than others. To make this more clear, we write in line 376-394:

> We presented different inputs to each *of ten pathways in random order*, corresponding to oriented bars in the visual cortex, or different single tone frequencies in the auditory cortex (Methods). We found that disinhibiting via the unspecific inhibitory population does not selectively potentiate E-to-E weights, and hence does not generate competition among the different inputs. *In this case, the selective potentiation of E-to-E weights corresponding to the inputs stimulated at a given time is counteracted by the potentiation of I-to-E weights specific to the stimulated inputs. This fast cancellation of any input-specific excitatory plasticity by input-specific inhibitory plasticity generates very small changes in the postsynaptic firing rate (Fig 7E, bottom).* In contrast, *equally* disinhibiting via all ten specific inhibitory populations strongly increases the E-to-E weights corresponding to only a subset of inputs, a process also called receptive field formation (Fig 7E). *In this case, the selective potentiation of E-to-E weights corresponding to the inputs stimulated at a given time is counteracted by the potentiation of all unspecific I-to-E weights. This leads to unbalanced excitatory and inhibitory plasticity due to the random presentation order of the different inputs thereby generating* stimulus-specific differences in excitatory and inhibitory inputs and hence leads to competition. *The input-specific potentiation is reflected in the fluctuating postsynaptic firing rate which increases only when the winning input is presented (Fig 7E, bottom).*

And we added to Methods line 842: "*(we set* $\rho^I_{spec} = -2$ *or* $\rho^I_{unsp} = -2$*)*".

Comment 11

Fig 6F please explain the connectivity. Were in this simulation the recurrently connected neurons added in place of the big triangle in the middle of 6D? In the scheme, there are multiple presynaptic neurons in each input, but only one postsynaptic. In 6F – do location of individual neurons and distance between them have any meaning? Or is it just to make the whole thing look like a brain (which is OK too), but please explain is there is a meaning.

Answer: In Fig 7F, each neuron in the recurrent circuit corresponds to a neuron that receives external input from ten different input streams as shown in Fig 7E. To make this clearer we added in line 395-397:

> Finally, we implemented a network of 30 recurrently connected excitatory neurons where each neuron *in the circuit* receives inputs from *ten* inputs and an unspecific and a specific inhibitory population *(as in Fig 7D).*

In Fig 7F, the location and distance of individual neurons does not have any meaning. We used the "digraph function" in Matlab which provides an automatic clustering of graphs, and brings similarly tuned neurons closer together in space. Besides making the detection of clusters easier, there is no further meaning in the distance

of individual neurons. To make this clearer, we added the following sentence in line 857-859 in the Methods section:

> The clustering graph in Fig 7F (left) was done with the digraph function in Matlab *where the distance between neurons is only used to visualize clusters of neurons with similar tuning.*

Furthermore, we added in the figure caption of Fig 7F:

> *Distance and position of neurons is for visualization purposes only.*

Reviewer #2:

Summary

The authors propose a novel nonlinear inhibitory plasticity rule that stabilises runaway excitation which arises from a nonlinear Hebbian excitatory plasticity rule. They use an approach based on the activity of populations of neurons and describe such plasticity rules based on the average firing-rate of the neurons of each population. This neuron model allows for elegant mathematical analyses to be made, which are complemented by simulations. The authors also define a sliding threshold for LTD and LTP in both excitatory and inhibitory plasticity rules which avoids extra fine tuning of parameters. As a result of the combination of both rules together, the authors show that a postsynaptic neuron forms a tuning curve when receiving independent inputs, which is diverse in a recurrent network, i.e., each neuron of the network responds preferentially to a distinct input feature after learning. The stability of synaptic dynamics is at the core of any type of learning and unveiling the nature of stabilising mechanisms are essential for us to understand how the brain learns (e.g., form memories). The authors propose an interesting new model of inhibitory plasticity which, differently than previous models, can stabilise excitatory plasticity while allowing for learning to still happen at excitatory synapses. Moreover, I like the analytical approach used in the manuscript which allow for conclusions to be drawn without extensive simulations and parameter sweeps. As interesting as these results are, however, I have major concerns about the relevance of the results when related to previous models of synaptic plasticity. Previous work, including the work by the authors have solved the so-called "temporal paradox" (as described by the authors in the Discussion section) with LTP/LTD sliding threshold (Bienenstock et al., 1982; Gjorgjieva et al., 2011; Zenke et al., 2013; Wu et al., 2020), normalization of weights (Litwin-Kumar and Doiron, 2014; Wu et al., 2020), and heterosynaptic plasticity (Zenke et al., 2015; Wu et al., 2020; Field et al., 2020; Kirchner and Gjorgjieva, 2021). I expand on this and other points below which I separated in major and minor points.

Major comments:

Comment 1

Comparison to previous models of excitatory plasticity. The excitatory synaptic plasticity model used by the authors (Eq. 1) resembles the one proposed by Leon Cooper in the 70s (Cooper, 1973; Cooper et al., 1979), which was modified later by Bienenstock, Cooper, and Munro (Bienenstock et al., 1982) to include a sliding-threshold for LTD/LTP. The sliding threshold does not only allow for selectivity to emerge, but also stabilises postsynaptic activity as shown more recently in spike-based versions of the BCM model (Gjorgjieva et al., 2011; Zenke et al., 2013). How would the BCM model (with sliding threshold) behave in the simulation paradigm used by the authors? Would an excitatory plasticity model with sliding threshold together with a linear inhibitory plasticity model be unstable? I would like to suggest repeating the simulations (and analysis) done in Fig 1 for a model with the sliding threshold as a comparison between the well established BCM model and the one used by the authors (maybe as a supplementary figure). Similar plasticity rules have been used by Litwin-Kumar and Doiron (2014) to study the formation of assemblies – excitatory learning rule similar to Eq. 1 and linear inhibitory plasticity model similar to Eq. 2. A comparison would be interesting (maybe in the discussion section). Additionally, how does this model relate to the previous model from Kirchner and Gjorgjieva (2021)

and Field et al. (2020)?

Answer: As pointed out by the reviewer, the BCM model has already been extensively studied in rate-based (e.g. [4, 38]) and spiking-based versions [14, 47] of networks with excitatory plasticity. This work has demonstrated that the BCM rule achieves weight selectivity and firing rate stability without any inhibitory plasticity. Adding inhibitory plasticity (linear or nonlinear) will not affect this, as long as the firing rate thresholds of excitatory and inhibitory plasticity overlap (see Fig 3A-C for what happens if they do not overlap). By allowing the sliding threshold c^E_{post} to evolve according to $\tau_c \frac{dc^E_{post}}{dt} = \frac{\nu^2}{\nu_{target}} - c^E_{post}$, where the target firing rate ν_{target} is equal to the LTD/LTP threshold of inhibition, i.e. $\nu_{target} = c^I_{post}$, the system remains stable independent of whether inhibitory plasticity is linear or nonlinear.

As the reviewer suggested, we repeated the simulations done in Fig 1, but with the BCM rule with a sliding threshold as defined above for excitatory plasticity and a linear inhibitory plasticity rule (Fig R1). As in our model (Fig 1), also here we found that initial conditions which where previously unstable, stabilized through the additional sliding threshold (Fig R1A,B). However, making the target rate unequal to the inhibitory LTD/LTP threshold, $\nu^E_{target} \neq c^I_{post}$, destabilized weight dynamics (Fig R1C), despite stable firing rates and sliding thresholds (Fig R1D). This is tightly linked to Fig 3 where we discussed the importance of overlapping LTD/LTP thresholds. We now extended the discussion of the BCM rule in the Discussion section to reflect these results. Here we additionally explain how our nonlinear inhibitory rule can implement a metaplastic mechanism of a sliding threshold.

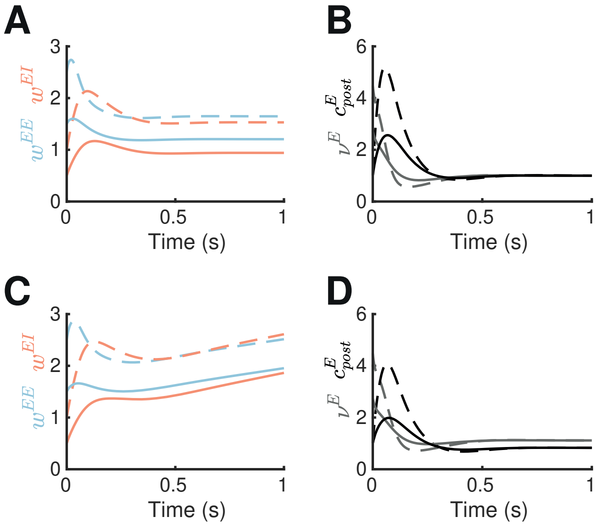

Figure R1: **The BCM rule with a sliding threshold for excitatory plasticity and linear inhibitory plasticity. A.** E-to-E (w^{EE}, blue) and I-to-E (w^{EI}, red) weights as a function of time for two initial conditions (solid lines, $[w^{EE}_0, w^{EI}_0] = [1.5, 0.5]$ and dashed lines, $[w^{EE}_0, w^{EI}_0] = [2.5, 1]$). Compare to Fig 1F. **B.** Postsynaptic firing rate (ν^E, gray) and sliding threshold (c^E_{post}, black) for initial conditions as in panel A. **C.** Same as A, but with $\nu^E_{target} \neq c^I_{post}$. Compare to Fig 1F and Fig 3A-C. **D.** Same as B, but with $\nu^E_{target} \neq c^I_{post}$.

Additionally, as the reviewer wrote, other modeling studies have successfully learned assembly structures (e.g. [26, 44, 42]) with similar learning mechanisms. Unlike our model, most of these studies have used additional mechanisms besides inhibitory and excitatory plasticity (even with the sliding threshold), including fast weight normalization, to reliably form assemblies (e.g. [26, 44, 42, 34]). Due to the temporal paradox, we proposed our nonlinear inhibitory plasticity rule as an alternative. We now also acknowledge that a different solution to the temporal paradox is heterosynaptic plasticity [10, 20], which the reviewer brought up (see also our response to Reviewer 1 Comment 3.)

We now add in the Introduction in lines 42-49:

> *For example, implementing metaplasticity in the plasticity of excitatory connections through a sliding threshold between potentiation and depression can generate weight selectivity and firing rate stability [4, 14, 47, 42]. Additionally, heterosynaptic plasticity has been modeled to stabilize synaptic weight dynamics, while still allowing learning [7, 39, 10, 20], including behavioral learning [6]. A strong candidate for stabilizing synaptic weights is the induction of homosynaptic LTP (LTD) together with heterosynaptic LTD (LTP) at nearby synapses, referred to as the 'Mexican hat' profile of homo- and heterosynaptic plasticity [41, 33].*

And in the discussion in lines 480-481:

> *Various implementations of the BCM rule have demonstrated its ability to achieve weight selectivity and firing rate stability without any inhibitory plasticity [4, 38, 14, 47].*

And in the discussion in lines 594-598:

> *The formation of strongly recurrently connected neurons, often referred to as assemblies, via synaptic plasticity has been shown in previous computational studies [26, 44, 30, 42, 34, 27]. In contrast to our framework, these studies rely on a fast normalization mechanism in addition to excitatory and inhibitory plasticity to reliably learn assemblies.*

Comment 2

Robustness to noise and time-varying inputs. The synaptic plasticity model used by the authors to describe changes in excitatory synapses has two fixed-points ($\dot{w}^{EE} = 0$) for the excitatory activity: $\nu^E = 0$ and $\nu^E = c^E_{post}$ according to Eq. 1. The first fixed-point ($\nu^E = 0$) is stable, and the second ($\nu^E = c^E_{post}$) is unstable as explained by the authors. The nonlinear inhibitory synaptic plasticity model proposed by the authors achieve stability by setting the postsynaptic firing-rate at $\nu^E = c^E$ post, i.e., the unstable fixed-point for excitatory plasticity. As long as $\nu^E = c^E$ post with machine precision (in the simulations), the system (postsynaptic neuron's activity) will remain constant. Would dynamical inputs (that change over time), or noise, result in excitatory weights drifting away from the unstable fixed-point, even though the nonlinear inhibitory plasticity mechanism may be constantly keeping $\nu^E \approx c^E$ post? It is not clear whether the system is stable when the input is constantly changing over time and/or noise is added to system. To test stability of this model, I would thus like to suggest two additional simulations. First, to add noise to the term $\nu^E(t)$, repeating the simulation from Fig 2C for longer periods of time, i.e., minutes or hours. Second, to use an input signal ρ^E that changes over time with, e.g, a sinusoidal signal.

Answer: We thank the reviewer for this very interesting comment. While preparing an answer for this question, we found an interesting difference in terms of weight stability by adding a varying input (either noise or sinusoid) to the presynaptic rate versus the postsynaptic rate. In short, adding noise or a sinusoidal input directly to the postsynaptic rate ν^E does not affect stability, while adding this variation to the presynaptic rate ρ^E leads to a drift of the synaptic weights to higher values.

We added a new Figure (Fig 6) and a new section titled "Performance of the nonlinear inhibitory plasticity rule under varying presynaptic input and postsynaptic firing rate" to the manuscript in lines 328ff, where we explain the differences between adding noise and varying inputs to presynaptic versus postsynaptic firing rates.

In addition, we added an additional subsection in the Methods to describe the model we used in more details (titled "Noise and sinusoidal input", see line 819ff) and we added an additional comment in the Discussion in lines 554-557:

> *Our framework is robust when noise or a varying input is added to the postsynaptic firing rate but not when the presynaptic rate varies (Fig 6). This suggests that additional homeostatic mechanisms are necessary to robustly counteract drift of synaptic weights when the input or the firing rates vary.*

Comment 3

Unstable initial conditions. Figs. 1E and 2B: the linear inhibitory plasticity model has a set of initial conditions (for w^{EE} and w^{EI}) that results in runaway excitation, while the nonlinear inhibitory plasticity model has a set of initial conditions that results in a silent postsynaptic neuron. Even though runaway excitation is presumably "worse" than no postsynaptic activity, the nonlinear inhibitory learning rule still fails to bring the postsynaptic activity to a steady non-zero firing-rate for a large set of initial conditions. This seems to me like a "weakness" of the nonlinear inhibitory plasticity model which is not mentioned in the text, apart from lines 672/673 in the Methods. Would it be beneficial to add a linear term to compensate for this? In this case the model would still be nonlinear but without any "forbidden" regions on the w^{EE} w^{EI} plane.

Answer: The reason for the region of initial conditions leading to zero firing rate in the case of the nonlinear inhibitory rule is that the rule induces no plasticity for zero postsynaptic rate (Fig 2A). This is consistent with experiments where nonzero postsynaptic firing rate, or postsynaptic depolarization, is required for induction of inhibitory plasticity [40]. In our model, depolarization corresponds to more excitatory than inhibitory input. This leads to a positive postsynaptic firing rate (based on the choice of our neuron model) and therefore, the induction of inhibitory plasticity.

In contrast, the linear inhibitory plasticity rule leads to inhibitory LTD even if the postsynaptic rate is zero. Whenever a neuron remains silent for some time, it will undergo persistent inhibitory LTD and eventually lose all of its incoming inhibitory synapses. When the neuron becomes active again after some time, it will fire with higher firing rates due to the absence of inhibitory input leading to runaway excitation. The suggestion to add a linear term to the current nonlinear rule will not resolve this problem.

Therefore, we suggest that the inability of the nonlinear inhibitory rule to make the postsynaptic firing rate non-zero for a large set of initial conditions is not a "weakness" but rather makes the rule more biologically plausible. We now write in the results in lines 187-191:

> Differently from the linear inhibitory plasticity rule (Eq 4), the nonlinear inhibitory plasticity rule ensures that I-to-E synapses do not change in the case where the postsynaptic firing rate is zero (Fig 2B, beyond gray line), as *shown in experiments where postsynaptic activity or depolarization is needed to induce inhibitory plasticity* [40].

Comment 4

Population versus individual neuron's dynamics. The authors use, for most of the analysis/simulations, a single population of presynaptic excitatory neurons, similarly for postsynaptic neurons and presynaptic inhibitory neurons (Figs. 1 to 5, and S1 Fig); $N^E = N^I = 1$. When both N^E and N^I are greater than 1, how does the activity of the different populations evolve over time? Fig 6E shows the evolution of weights, but it's unclear how the different populations' activities evolve. Additionally, the authors claim that because the set-points c^E_{post} and c^I_{post} reach a steady state for single populations ($N^E = N^I = 1$), it is safe to assume that they will have constant values. However, it's not clear to me what happens when both N^E and N^I are greater than one if the set-points are also evolving over time. I would like to suggest confirming this claim by running simulations, such as the ones described in Fig 6, with both set-points evolving over time.

Answer: We will answer this question in three parts. First, in Fig 1-6 and S2 Fig the postsynaptic neuron receives input from a single excitatory input population with N^E neurons and a single inhibitory population with N^I neurons (see for e.g. the caption of Fig 1A). Only in Fig 7 we use multiple input populations, and then N^E and N^I still represent the number of excitatory and inhibitory neurons per population. Since we assume that all neurons are the same (we are doing mean-field), we can drop the subscripts j and k in Eq 1 and Eq 2 and compute the total excitatory input to the postsynaptic neuron as $N^E \rho^E w^{EE}$ and the total inhibitory input as $N^I \nu^I w^{EI}$. Therefore, the number of neurons becomes a multiplicative factor in the total excitatory and inhibitory input to the postsynaptic neuron. How exactly N^E and N^I affect our results is explicitly shown in the equations (see Eq 6-9) and described in the Methods section. Indeed, for the simulations in Fig 1-6 and S2

Fig we used $N^E = 1$ and $N^I = 1$, but this choice was completely arbitrary, and any other choice would just scale the relevant variables (the postsynaptic firing rate and the weights) without affecting the results.

The second question pertains to how the activity of different populations evolves over time. We only model a single postsynaptic neuron driven by one (Fig 1-6) or multiple (Fig 7A-E) input populations, which all have fixed firing rates (except Fig 6). Therefore, only the firing rate of the postsynaptic neuron varies as the weights evolve over time. We have now added the firing rate of the postsynaptic neuron to Fig 7E, where we used 10 input populations, and two inhibitory populations, one specific and one unspecific (see Fig 7D-F and Table 2). Perturbing the excitatory inputs also affects the inhibitory firing rates (as shown in Fig 4B). Since the perturbations in Fig 7A-C are similar to Fig 4, we decided to not show the inhibitory firing rates in Fig 7A-C.

Lastly, to answer the final reviewer's question we tested our proposed dynamic matching of the excitatory and inhibitory postsynaptic thresholds (Fig 3D-F; Eq. 22) for two input populations, repeating the analysis from Fig 7B (S3 Fig G-I). We implemented a global threshold parameter which dynamically matches the postsynaptic thresholds c^E_{post} and c^I_{post} to the same value for each input (S3 Fig H) and achieves stable weight dynamics and activity (S3 Fig I). Therefore, the proposed dynamic threshold matching also achieves stability when using multiple input populations. However, this model (using the parameters from Fig 7D) disrupts the formation of receptive fields since it does not induce competition among the different input streams. As we discuss also in response to Comment 6, we feel that this is beyond the scope of the current work and leave it to future work to investigate how different implementations of dynamic threshold mechanisms can form stable receptive fields and recurrent structures.

We added in the discussion in lines 536-543:

> *A limitation of the suggested dynamic threshold matching mechanism is that it is non-local whereby the thresholds for all input pathways converge to the same value. While this can still achieve stable weight dynamics and postsynaptic firing rates (S3 Fig G-I; Methods), it can no longer induce competition among different inputs. Future work needs to investigate whether a different dynamic matching of excitatory and inhibitory LTD/LTP thresholds, perhaps one that is input-specific, can achieve the stable formation of receptive fields.*

In the Methods we add in line 773-776:

> *For multiple input streams (S3 Fig G-I), the dynamic postsynaptic LTD/LTP thresholds change based on the total excitatory (or inhibitory) weight change, leading to a non-local sliding mechanism which is independent of the input stream.*

Comment 5

Neuron model. Many of the results, and consequently, conclusions of the manuscript rely on the choice of the neuron model (Eqs. 7 and 8). Using a linear rectifier as a model to describe the firing-rate of the neurons is a valid choice, especially for mathematical tractability. Nevertheless, most of the results depend on this choice. For example, the dependence of excitatory plasticity on the presynaptic rate and inhibitory-to-excitatory weights (shown, e.g., in Figs. 1C and 4E) is indirect: excitatory plasticity depends only on ρ^E and ν^E directly (Eq. 1). For example, in Fig 1C the different curves assigned to different w^{EI} reflect changes caused by distinct ν^E that are a consequence of changing w^{EI} while keeping the other parameters (ρ^E, ρ^I, N^E, N^I , and w^{IE}) fixed. To clarify the effect of these parameters I would like to suggest including Eqs. 7 and 8 in the main text so that it's easier for a reader to understand how changes in, e.g., w^{EI} , affect the curves in Fig 1C. Additionally, it would be interesting to have a 3D color plot (such as the one in Fig 6E) showing the changes in weight ($\dot{w}^{EE}$, color) as a function of ρ^E (e.g., x-axis) and ν^E (e.g., y-axis).

Answer: Thanks for this good suggestion. As proposed, we have now added the neuron model for excitation and inhibition (former Eq 7 and 8) into the main text (now Eq 1 and 2) at lines 92-105. Furthermore, we have added a new supplementary figure (S1 Fig) which shows the weight change as a function of pre- and postsynaptic activity. We refer to the new supplementary figure in the text in line 115.

Comment 6

Assuming static c^E_{post} and c^I_{post} (from page 9 onward). As mentioned above (point 4), the assumption that the dynamics given by Eq. 20 will lead to constant c^E_{post} and c^I_{post} for the case involving multiple populations might not hold true. I would like to suggest carefully checking if that assumption holds, especially in case noise or varying inputs are used. Additionally, the dynamics proposed for c^E_{post} and c^I_{post} seem to create a plasticity mechanism that is non-local (Eq. 20). Could the authors clarify why they chose to not use the classic BCM sliding threshold?

Answer: We agree with the reviewer about carefully exploring the conditions under which the dynamic thresholds lead to a stable configuration. To address Reviewer 3 Comment 1-3, we have added a new supplementary figure to the manuscript (S3 Fig). We show that starting from other initial configurations of the excitatory and inhibitory thresholds (e.g. $c^I_{post} < c^E_{post}$) leads to matched thresholds and stabilization of rate and weight dynamics (S3 Fig A-C). Furthermore, we verify that performing perturbations (as we have done previously in Fig 4), while the thresholds are dynamic, leads to stable configurations (S3 Fig D-F).

In Fig R2, we show that adding postsynaptic noise and varying input (sinusoid) still leads to stable weight, rate and threshold dynamics. Since the dynamic matching of thresholds is not a main point of our paper, we decided to not include this figure in the main manuscript. We have addressed the effect of adding noise and varying inputs in detail in response to Comment 2 (and new Figure 6), and dynamic threshold matching under perturbation (new S3 Figure).

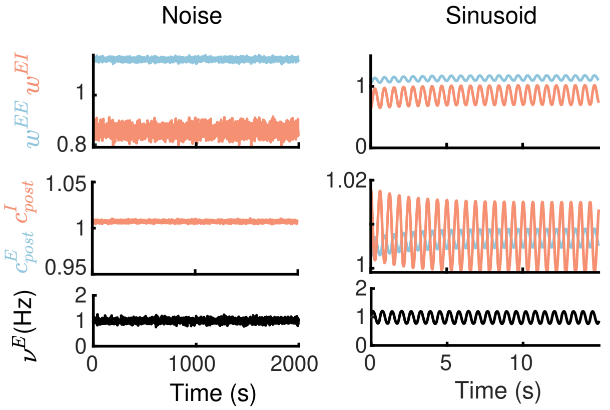

Figure R2: **Dynamic excitatory and inhibitory LTD/LTP thresholds plus postsynaptic noise (left) and sinusiodal input (right).** Top: E-to-E (w^{EE}, blue) and I-to-E (w^{EI}, red) as a function of time. Middle: Excitatory (c^E_{post}, blue) and inhibitory (c^I_{post}, red) postsynaptic LTD/LTP threshold as a function of time. Bottom: Postsynaptic rate dynamics (ν^E, black) as a function of time.

Why did we not choose the classic BCM sliding threshold? Although the BCM framework has been successfully applied to form stable receptive fields in different scenarios (see e.g. [8]), one disadvantage is that it can lead to strong oscillatory behavior [35]. Our dynamic threshold mechanism is an alternative suggestion to the classical sliding threshold in the BCM rule. We acknowledge the limitation of this mechanism in line 536-543 in the Discussion:

> *A limitation of the suggested dynamic threshold matching mechanism is that it is non-local whereby the thresholds for all input pathways converge to the same value. While this can still achieve stable weight dynamics and postsynaptic firing rates (S3 Fig G-I; Methods), it can no longer induce competition among different inputs. Future work needs to investigate whether a different dynamic matching of excitatory and inhibitory LTD/LTP thresholds, perhaps one that is input-specific, can achieve the stable formation of receptive fields.*

Comment 7

Fixed E/I ratio. The authors define E/I ratio as $R^{E/I} = w^{EE}/w^{EI}$ and show that this quantity is regulated by the combination of excitatory and inhibitory nonlinear plasticity rules (Eqs. 1 and 3). They calculate this quantity (Eq. 6) and state that "The nonlinear inhibitory plasticity rule establishes a fixed excitatory and inhibitory weight ratio". In what sense is it fixed? I would consider as "fixed" if the value is independent of, e.g., inputs, and thus being a constant. The fact that it depends on the level of excitatory and inhibitory inputs means that it's not fixed; is that correct? Additionally, the typical definition of E/I balance is related to input currents. In this neuron model this would be $E/I = (w^{EE}\rho^E)/(w^{EI}\nu^I)$. What happens with this quantity? The authors claim that the combination of the nonlinear excitatory and inhibitory plasticity model (Eqs. 1 and 3) create an E/I set-point. However, as shown in Figs. 2D and 5A, the E/I ratio is defined by a line attractor instead of a single set-point. I would like to suggest clarifying this in the text.

Answer: To acknowledge that the E/I ratio indeed depends on other variables, e.g. the excitatory input firing rate ρ^E (as shown in Fig 5A), we now use the term "set-point" instead of "fixed" in the entire text (Results and Discussion sections, see for e.g. lines 546-552). What we originally meant by a "fixed" E/I ratio was that for fixed parameters (e.g. excitatory input rates) the interaction of excitatory and inhibitory plasticity will always lead to the same E/I ratio, independent of the initial conditions, in the limit of large I-to-E weights (as shown in Eq 9 and explained in Fig 5). We added in the Results section, line 307:

> *We refer to Eq 9 as the E/I ratio set-point for fixed input rates.*

Regarding the definition of E/I balance, we could have indeed chosen the ratio of the total excitatory divided by the total inhibitory input $\tilde{R}^{E/I} = (N^E w^{EE}\rho^E)/(N^I w^{EI}\nu^I)$ (as suggested by the reviewer). However, to calculate the E/I ratio we assume that the firing rates reach a steady state; hence, the difference between the weight E/I ratio $R^{E/I}$ and the "total" E/I ratio $\tilde{R}^{E/I}$ is multiplicative ($\tilde{R}^{E/I} = R^{E/I} N^E \rho^E / \left(N^I \nu^I\right)$). Therefore, the interpretation of our results in Fig 5 remains the same, since the firing rates ρ^E and ν^I are considered to be at steady state. We now state the alternative definition of the E/I balance in the Methods section in line 813-818:

> *The E/I balance can also be defined by the total excitatory input divided by the total inhibitory input onto the postsynaptic neuron:*
>
> $$R^{E/I}_{tot} = \left(N^E w^{EE} \rho^E\right) / \left(N^I w^{EI} \nu^I\right). \tag{R1}$$
>
> *This leads to:*
>
> $$R^{E/I}_{tot} = \left(N^I (N^E \rho^E w^{IE} + \rho^I) w^{EI} + c_{post}\right) / \left(N^I (N^E \rho^E w^{IE} + \rho^I) w^{EI}\right). \tag{R2}$$
>
> *However, since we calculate the E/I balance at steady state, the total E/I balance is equal to the weight E/I balance multiplied by a constant*
>
> $$\tilde{R}^{E/I} = R^{E/I} N^E \rho^E / (N^I \nu^I). \tag{R3}$$
>
> *Therefore, the results in Fig 5 also hold with this alternative E/I ratio definition.*

Finally, we answer the reviewer's question on how the line attractor is connected to the E/I ratio. The phase plane in Fig 2B shows that the line attractor defines the I-to-E weight w^{EI} as a multiple of the E-to-E weight w^{EE} with the multiplying factor $N^E\rho^E/(N^I\nu^I)$ (the slope of the line attractor) minus an offset term $c_{post}/(N^I\nu^I)$ (see also Eq 7 and Fig 2D). The ratio of excitatory to inhibitory weight strengths, $R^{E/I}$ (Eq 8), can be expressed as a sum of two terms: one constant term equal to the slope of the line attractor, which is independent of w^{EE} and w^{EI}, and a second term, called an offset, which depends on w^{EI}. For sufficiently large w^{EI}, the offset term can be ignored so that the E/I ratio set-point, $R^{E/I}_{\infty}$, becomes independent from w^{EE} and w^{EI} and equal to the slope of the line attractor. We now explain in the Methods in line 787-794:

The existence of an E/I ratio set-point can be directly related to the line attractor. The line attractor (Eq 7) expresses the I-to-E weight w^{EI} as a multiple of the E-to-E weight w^{EE} minus the offset term $c_{post}/(N^I \nu^I)$. Therefore, the ratio of excitatory to inhibitory weight strengths, $R^{E/I}$ (Eq 8), can be expressed as the sum of two terms: one constant term equal to the slope of the line attractor, which is independent of the E-to-E and I-to-E weights, w^{EE} and w^{EI}, and a second term, called an offset, which depends on w^{EI}. When this weight is sufficiently large, the offset term can be ignored, leading to an E/I ratio set-point, $R_{\infty}^{E/I}$, independent from the E-to-E and I-to-E weights.

and have added in lines 312-314:

> Analytically, this corresponds to a line attractor with a steeper slope (Fig 2D and Fig 4D for increasing ρ^E) *since the E/I ratio set-point* $R_{\infty}^{E/I}$ *corresponds to the slope of the line attractor (Fig 2D; Methods).*

Minor points:

Comment 8

Abstract:"Synaptic changes underlie learning and memory formation in the brain." I suggest adding the word "hypothesised" here.

> **Answer:** We added the word.

Comment 9

Abstract: "But synaptic plasticity of excitatory synapses on its own is unstable, ..." This is sentence is inaccurate if synaptic plasticity of excitatory synapses is defined as any mechanism that is involved in modifications of excitatory synapses, e.g., synaptic scaling (Wu et al., 2020). Are the authors referring here to Hebbian plasticity? I suggest rephrasing this sentence.

> **Answer:** Indeed, we refer to Hebbian excitatory plasticity. We clarify this now.

Comment 10

Abstract: "... leading to unlimited growth of synaptic strengths without additional homeostatic mechanisms." Following the point above (point 9) – is that related to Hebbian plasticity of excitatory synapses? In this case I would like to point-out to an additional undesirable state, which is silence, i.e., zero firing-rate. In this case Hebbian plasticity of excitatory synapses can lead to either runaway excitation or complete silence. I suggest rephrasing and clarifying this sentence.

> **Answer:** We added the point that silencing the neuronal activity is also an undesirable case.

Comment 11

Abstract: "We identify two key features of inhibitory plasticity, dominance of inhibition over excitation and a nonlinear dependence on the firing rate of postsynaptic excitatory neurons whereby inhibitory synaptic strengths change in the same direction as excitatory synaptic strengths." It seems that the nonlinear dependence on the firing-rate of postsynaptic excitatory neurons is explicit from the model, and it is thus not a key feature identified by the authors, but a feature imposed by the authors. I would like to suggest rephrasing this sentence. The last part of the sentence is confusing to me ("whereby inhibitory synaptic strengths change in the same direction as excitatory synaptic strengths.") Is this related to dominance of inhibition over excitation or nonlinear dependence on the firing rate of postsynaptic excitatory neurons?

Answer: We replaced the word "identified" with the word "suggest". We also clarified what we meant by the term "same direction".

Comment 12

Abstract: "... by this novel inhibitory plasticity achieve ..." I would like to suggest adding the word model after plasticity.

Answer: We added the word.

Comment 13

Abstract: "... achieve a fixed excitatory/inhibitory set-point in agreement with experimental results." The excitatory/inhibitory set-point referred here is for weights and not currents as shown experimentally. I would like to suggest rephrasing this sentence to accurately describing the results from the model.

Answer: We added the word "weight" so it is clear that it is the E/I weight set-point.

Comment 14

Line 2: "Learning and memory formation in the brain are implemented by synaptic changes" I would like to suggest adding the word hypothesis or theorised, as references 1 and 2 are not confirmation of this claim but hypotheses/theories.

Answer: We added the term "hypothesized".

Comment 15

Line 77: "Here, a population of presynaptic excitatory neurons projects to a population of inhibitory neurons and both populations project to a postsynaptic excitatory neuron." The mean-field type of modelling (e.g., Eqs. 1, 2, and 3) typically assumes average activity of a population being described by the variables. Could the authors clarify why ν^E describes a single neuron and not a population?

Answer: The reviewer is correct that we used a mean-field approach and hence the excitatory and inhibitory input is each modeled as a population of neurons. The same could indeed have been done for the postsynaptic neuron (i.e. use a population of postsynaptic neurons). The reason we used a single postsynaptic neuron was to make it clear that we study the plasticity of feedforward connections: from the excitatory input population directly to the postsynaptic neuron and indirectly through the inhibitory input population. This approach is common for other computational models, including many using the BCM rule. Had we used a postsynaptic population, we might have had to describe a mean-field model for the recurrent connectivity within the population of postsynaptic neurons. We agree that this is very interesting, however, solving mean-field equations in recurrent networks and deriving synaptic plasticity requires different kinds of calculations and we decided to leave this for a future publication.

Comment 16

Eq. 1: Are the units correct? From Eq. 7 it looks like w^{EE} is unit-free, thus the left side of Eq. 1 is unit-free given that the unit of τ_w^E is milliseconds (representing time). The variables ρ^E, ν^E, and c_{post}^E have the same unit, Hz, so the right side of Eq. 1 has the unit of Hz 3 . Wouldn't it be better to use a learning rate with a correcting unit instead of a characteristic time?

Answer: The reviewer is right, the units were not correct. Instead of using an additional parameter for the learning rate, we now modified the time constants to have units of Hz^2 for the nonlinear learning rule and Hz

for the linear learning rule (see Table 1). Hence, the taus can be thought of as the inverse of the learning rates. We now make a comment of this in the Methods.

Comment 17

Line 100: "In our framework, inhibitory neurons can affect excitatory plasticity in three equivalent ways." These three ways reflect the author's choice of neuron model (Eqs. 7 and 8). I would like to suggest including these two equations in the main text to explicitly show how the neuron model affects the plasticity rule. This way it's easy to follow that, e.g., $\nu^E = \rho^E w^{EE} - (\rho^E w^{IE} + \rho^I) w^{EI}$ for $N^E = N^I = 1$ (from the steady states of Eq. 7 and 8).

Answer: We have followed the reviewer's suggestion and shifted the respective equations to the Results section, which are now Eq. 1 and Eq. 2, see lines 92-105.

Comment 18

Eq. 4: The authors derived this equation considering the steady-state of ν^E. Could the authors do the same for ν^I to write the left side of the inequality as $N^I(N^E\rho^E w^{IE} + \rho^I)^2/\tau_w^I$?

Answer: Yes, we can assume in addition to ν^E, ν^I is also at steady state. This allows us to write the inequality as suggested by the reviewer because $\nu^I = N^E\rho^E w^{IE} + \rho^I$. We added this information in line 753 in the Methods section and explicitly show the inequality with $\nu^I = N^E\rho^E w^{IE} + \rho^I$ in Eq 21.

Comment 19

Eq. 5: Similar to the point above (point 18). Could the authors rewrite the right side of the equation using the steady state of ν^I?

Answer: Yes, ν^I can be replaced by its steady state value also in Eq 7 (Eq 5 in the manuscript in our first submission). We now mention in the Methods section and we explicitly show the line attractor with $\nu^I = N^E\rho^E w^{IE} + \rho^I$ in Eq 22.

Comment 20

Line 202/203: "When the excitatory postsynaptic threshold is lower than the inhibitory postsynaptic threshold ($c^E_{post} < c^I_post$)..." I would like to suggest adding here what happens in the other case, i.e., when $c^E_{post} > c^I_post$.

Answer: We agree with the reviewer, that it was not clear in our manuscript what happens if $c^E_{post} > c^I_post$. We now explicitly show this in our new supplementary figure (S3 Fig A-C), where $c^I_{post,0} = 0.7$ and $c^E_{post,0} = 1.3$ (flipped case from Fig 3D-F). The thresholds eventually converge to the same value (S3 Fig B), while the synaptic weights and the postsynaptic firing rate stabilize (S3 Fig C).

We added in lines 246-249 in the results section:

> *The excitatory and inhibitory LTD/LTP thresholds can be matched, as well as rates and weights stabilized also for other initializations of the LTD/LTP thresholds (S3 Fig A-C).*

Please also see our answer to Reviewer 3 Comment 2.

Comment 21

Line 210/211: "Motivated by experimental findings and theoretical considerations [44], we proposed that these thresholds can be dynamically regulated in opposite directions (Fig 3D; see Methods). When the postsynaptic rate is lower than the excitatory postsynaptic LTD/LTP threshold ($\nu^E > c^E_{post}$), the excitatory postsynaptic

LTD/LTP threshold should decrease, while when the postsynaptic rate is higher than the threshold ($\nu^E < c^E_{post}$), the excitatory threshold should increase." For the best of my knowledge, the description of the threshold dynamics for excitatory plasticity is exactly how the BCM theory was first described (Bienenstock et al., 1982). I would like to suggest rephrasing this first sentence as the authors are not proposing a new form of sliding threshold, but proposing that inhibitory plasticity also has one.

Answer: While a sliding mechanism of the excitatory threshold has already been suggested for the BCM rule, our suggested mechanism is different in two ways. First, we propose that also the inhibitory threshold is dynamic and can slide, and second, that the excitatory and inhibitory thresholds slide into opposite directions. To clarify, we now write in lines 234-237:

> Motivated by experimental findings and theoretical considerations *that the excitatory threshold can slide [18, 4], here we proposed that the inhibitory threshold can also be dynamically regulated with both excitatory and inhibitory thresholds shifting into* opposite directions (Fig 3D; see Methods).

Comment 22

Line 219: "... these dynamic ..." The letter s is missing: dynamics.

Answer: We added the missing letter.

Comment 23

Line 224: "The generation of such heterogeneous postsynaptic rates is consistent with experimental observations in multiple brain regions [45]." It looks like the postsynaptic firing-rate is either $\nu^E \approx 0.95Hz$ or $\nu^E \approx 0.99Hz$ in Fig 3F. The statement that this constitutes a heterogeneous postsynaptic rate that is consistent with experimental findings is a bit too strong. I would like to suggest adding more simulations (or mathematical analysis) to show heterogeneous firing-rate in, e.g., a histogram plot of firing-rates or to change this sentence.

Answer: We agree with the reviewer that our statement was imprecise. Indeed, typically, such heterogeneity refers to the firing rates that can be reached in different neurons in an entire population. But in our model we have a single postsynaptic neuron, and what we actually meant was that this neuron can achieve a wide range of firing rates. Starting from different initial conditions for the postsynaptic LTD/LTP thresholds, $c^E_{post,0}$ and $c^I_{post,0}$, the rule will match them somewhere between their initial values so that they ultimately converge to different final values, $c^E_{post} = c^I_{post}$ (see response to Reviewer 1 Comment 1-3). Therefore, in principle, our dynamic threshold matching can realize any postsynapitc firing rate. We wanted to establish that this is different to many implementations of the BCM rule, which imposes a target postsynaptic firing rate independent of initial conditions (please see our answer to Comment 45). We now write in line 251-252:

> *Therefore, for different initializations of the LTD/LTP thresholds, a wide variety of stable postsynaptic rates is possible.*

Comment 24

Line 228/229: "Since even if they are unequal, excitatory and inhibitory LTD/LTP thresholds can be dynamically matched, from now on we assumed that they are equal and static." Please see point 6.

Answer: Please see our answer to Comment 6. We have now modified the sentence as:

> Excitatory and inhibitory LTD/LTP thresholds can be dynamically matched *under most conditions, even if they are unequal (S3 Fig). Therefore,* from now on we assumed that they are equal and static (as shown in Fig 2A).

Comment 25

Line 242-246: "For example, it has been shown that input perturbations ..." I would like to suggest moving these sentences to the Discussion section as it does not present new results but discuss them in the scope of previous work.

Answer: We have moved the respective sentences to the Discussion section, lines 500-507.

Comment 26

Line 247-250: "Our framework can even predict the steady values ..." This is only true for the specific choice of neuron model. I would like to suggest rephrasing this sentence so that it's clear that it depends on the neuron model (see point 5).

Answer: We have followed the reviewer's suggestion and now write:

> *Since we used a threshold-linear neuron model (Eq 1,2),* our framework can even predict the steady values of the E-to-E and I-to-E synaptic weights, as well as their ratio, by calculating the line attractor in the phase space of w^{EE} and w^{EI} weights as a function of the perturbed parameter (Fig 4D).

Comment 27

Line 256/257: "Such a shift in the plasticity threshold for excitatory synapses has been measured ..." The threshold that changes in the model is for the presynaptic activity (Fig 4E). Is it the same for the experimental results cited here?

Answer: Yes, the experimental references presented here refer to LTD/LTP threshold change based on presynaptic activity. To clarify this we write in line 283-287:

> Such a shift in the plasticity threshold for excitatory synapses *based on presynaptic activity* has been measured in sensory deprivation experiments [21, 32, 24], and while restoring vision after sensory deprivation [32, 8] *(although deprivation-induced effects occur on much slower timescales than in our plasticity model, see Discussion).*

Comment 28

Eq. 6: This equation is valid for the neuron model used here (see point 5). I would like to suggest adding this to the text. Additionally (see point 7), the excitatory-to-inhibitory ratio (E/I) is usually calculated based on input currents, which would be $R^{E/I} = \frac{\rho^E w^{EE}}{\nu^I w^{EI}}$ with the neuron model used by the authors (Eq. 7).

Answer: We have now added the information about our neuron model in line 302-303:

> Given our ability to calculate the steady states of the weights *having used a linearly rectified neuron model* (Fig 4D), we studied the ratio of E-to-E and I-to-E weights:

We answer the issue on the different definition of E/I ratios in our response to comment 7.

Comment 29

Eq. 6: To arrive in this equations the system is in a steady state, and thus $\nu^I = N^E w^{IE} \rho^E + \rho^I$, which results in $R^{E/I} = \frac{N^I (N^E \rho^E w^{IE} + \rho^I) w^{EI} + c_{post}}{N^E \rho^E w^{EI}}$. I would like to suggest explicitly showing the calculation considering the parameters of the neuron model.

Answer: As suggested by the reviewer, we now explicitly show this equation in line 303, Eq 8).

Comment 30

Line 273: "For strong I-to-E weights w^{EI} , the E/I ratio approximates to $R_{\infty}^{E/I}$ ..." This condition is valid for $N^I \nu^I w^{EI} \gg c_{post}$ according to Eq. 6. Given that the steady state of ν^I can be calculated analytically, this expression becomes $N^I(N^E \rho^E w^{IE} + \rho^I)w^{EI} \gg c_{post}$. I would like to suggest clarifying the contribution of the parameters of the model, especially ρ^E, N^E, w^{IE}, and ρ^I.

Answer: We explicitly discuss this condition and its dependence on the parameters now in line 782-786. We write:

> *This derivation is only valid for $N^I(N^E \rho^E w^{IE} + \rho^I)w^{EI} \gg c_{post}$. Therefore, the parameters of the input firing rates ρ^E and ρ^I, the synaptic weights w^{EI} and w^{IE}, as well as number of excitatory and inhibitory neurons N^E and N^I need to be chosen appropriately. This inequality is satisfied for the parameters in Fig 5 (Table 1).*

Comment 31

Line 273: "... E/I ratio approximates to $R_{\infty}^{E/I} = \frac{N^I \nu^I}{N^E \rho^E}$..." Considering the steady state for ν^I, this term becomes $R_{\infty}^{E/I} = \frac{N^I(N^E \rho^E w^{IE} + \rho^I)}{N^E \rho^E}$. I would like to suggest adding this extra step to clarify how the neuron model's parameters influence the ratio E/I.

Answer: As suggested by the reviewer, we added the extra step in line 305, Eq 9.

Comment 32

Line 278/279: "... which needs to be counteracted by even more inhibitory LTP to stabilize the weights." Wouldn't it be to stabilize the weight dynamics?

Answer: Yes, the reviewer is correct. To clarify we now write in line 311: "...which *is counteracted by even more inhibitory LTP to stabilize weight dynamics.*"

Comment 33

Line 284/285: "matching experiments [26]." Which experiments do the authors refer to?

Answer: We now write in line 318-320 in the results: "...matching experiments *in the mouse auditory cortex where inducing excitatory and inhibitory plasticity generates an E/I ratio set-point* [9]." Please also see our response to Reviewer 3 Comment 5.

Comment 34

Line 311/312: "we applied a disinhibitory signal by decreasing the external excitatory input onto the inhibitory populations." I could not find an equation related to this implementation in the Methods. Considering Eqs. 7 and 8, changes in the excitatory input, ρ^E , affect both ν^E and ν^I. I would like to suggest adding a variable to Eq. 8 to indicate how disinhibition is implemented, and the parameter used for this variable in Fig. 6.

Answer: At the time point of disinhibition, we change the value of the external input onto the inhibitory neurons to -2. We now clarify this in line 842 in the Methods: "*(we set $\rho^I_{spec} = -2$ or $\rho^I_{unsp} = -2$)*". We also added this information to the caption of Fig 7:

> Purple bars indicate the time window where either the unspecific (yellow) or all specific (red) inhibitory populations is disinhibited *by applying a negative input onto the inhibitory neurons* (Methods).

Comment 35

Lines 351 to 354: Aren't these "two key features" strictly necessary for the stability?

Answer: These two features are indeed necessary, we added the word "necessary" in line 416.

Comment 36

Line 355: "... with a suitable mechanism that enables self-adjusting of the plasticity thresholds ..." Doesn't this mechanism make the plasticity rules non-local? It would be worth mentioning this in the discussion.

Answer: Yes, the reviewer is correct that our suggested mechanism is non-local. We have now added new text in the Discussion in lines 536-543 (see also our response to Comments 4, 6 and 45) and in the Methods in lines 773-776:

> *For multiple input streams (S3 Fig G-I), the dynamic postsynaptic LTD/LTP thresholds change based on the total excitatory (or inhibitory) weight change, leading to a non-local sliding mechanism which is independent of the input stream.*

Comment 37

Line 360: ".. in agreement with experiments [26]." Which experiments? Could the authors elaborate on this?

Answer: We included more information in the Discussion in lines 408-516 (as noted in our response to Comment 33). Please see also our answer to Reviewer 3 Comment 5, where we state in multiple positions in the text the link between our model and experimental results.

Comment 38

Lines 372 to 374: "However, the spatial and temporal scales for integrating Hebbian and homeostatic plasticity have remained an open question [17,20,21]." Isn't heterosynaptic plasticity (Zenke et al., 2015; Field et al., 2020; Kirchner and Gjorgjieva, 2021) a solution for this question?

Answer: The reviewer is correct, we have now made this more explicit by writing in the Discussion in lines 434-445:

> *At the same time, computational studies have included multiple homeostatic mechanisms, some of them the same as the experimental ones, to stabilize rates and weight dynamics, including upper bounds on the E-to-E weights, normalization mechanisms [31, 29, 26, 44, 42, 34], metaplastic changes of the plasticity function [4, 14, 47, 43, 42], heterosynaptic plasticity [39, 10, 20] and intrinsic plasticity and synaptic scaling [42].* However, the spatial and temporal scales for integrating Hebbian and homeostatic plasticity have remained an open question [37, 46, 45]. *This is especially the case for synaptic scaling which experimentally operates on timescales too slow to counteract the faster Hebbian synaptic plasticity (hours and days, vs. seconds). Heterosynaptic plasticity is instead a more natural solution to the 'temporal paradox' problem since it operates on a similar timescale as Hebbian plasticity [7, 39, 10].*

Please also see our answer to Comment 1 above, and our answer to Reviewer 1 Comment 2 and 3 for more details on this point.

Comment 39

Line 424 to 426: "More dominant means that I-to-E weights need to change with a higher magnitude at each time step compared to E-to-E weights, for all postsynaptic rates." Isn't this finding contradictory to the experimental results from Froemke et al. (2007); particularly Fig. 4 from this experimental paper?

Answer: It is true that in Fig 4 in Froemke et al., (2007) the excitatory receptive field is first remapped while the inhibitory receptive field follows with a delay of around 100 minutes, suggesting that excitatory plasticity operates faster than inhibitory plasticity. However, in this work plasticity is induced by pairing nucleus basalis activation with pure tone frequencies, which shifts the excitatory and inhibitory tuning profile for a pure tone stimulus. A more direct evidence for our hypothesis that "I-to-E weights need to change with a higher magnitude at each time step compared to E-to-E weights" follows from findings in D'Amour and Froemke 2015. In their Fig 7G and 7J, inhibitory synapses change more drastically than excitatory synapses in a pairing protocol. We now write in line 527-529 in the Discussion:

> *Previous experimental work has reported that inhibitory synapses change more drastically than excitatory synapses [9], but inhibitory plasticity may be delayed relative to excitatory plasticity [11].*

Comment 40

Lines 450 to 453: "This is consistent with several experimental studies which have suggested that inhibitory plasticity keeps an E/I ratio set-point [9,26,32,36,62,80–83]." Isn't the set-point for both excitatory and inhibitory rules defined by the postsynaptic firing-rate, ν^E (Eqs. 1 and 3)? Additionally, from Eq. 6, the E/I ratio is described as a result from other variables, such as ρ^E, and thus changes according to the presynaptic firing-rate – in this case it would not be a set-point, but a by-product of the firing-rate set-point.

Answer: The E/I ratio set-point is a direct consequence of the overlap of the LTD/LTP thresholds of excitatory and inhibitory synaptic plasticity, and hence, is determined by the postsynaptic firing rate (which is exactly at the LTP/LTD threshold). We now write in lines 559-561:

> *The interaction of the nonlinear inhibitory and excitatory plasticity in our model and the overlap of excitatory and inhibitory LTD/LTP thresholds* lead to an E/I weight ratio set-point (Fig 5A,C and Eq 8).

The set-point is calculated under the steady-state assumption where all variables are constant. Changing any one of these variables (e.g. the input rate ρ^E) will lead to a different set-point (which we show in Fig 7B and explain in the context of Eq. 6). Please also see our answer to comment 7.

Comment 41

Line 461/462: "The emergence of an E/I ratio set-point and the stabilization of rates driven by the novel inhibitory plasticity rule ensure a fixed E/I balance" See points above.

Answer: We modified this to (line 572): "The emergence of an E/I ratio set-point *following from* the stabilization of rates driven by the novel inhibitory plasticity rule ensures E/I balance."

Comment 42

Line 552 to 554: "Functionally, our proposed inhibitory plasticity can establish and maintain a fixed E/I ratio set-point. At this set-point, no synaptic plasticity is induced, i.e. plasticity is "off"." According to Eqs. 1 and 3, plasticity is off when $\nu^E = c^E_{post} = c^I_{post}$, which happens for specific combinations w^{EE} and w^{EI}. I would like to suggest rephrasing these sentences to clarify that it's not the E/I ratio set-point that switches plasticity off, but the firing-rate set-point.

Answer: We clarified this now in lines 669-672:

> Functionally, our proposed inhibitory plasticity can establish and maintain an E/I ratio set-point *at which the postsynaptic firing rate is exactly at the LTD/LTP threshold. For such postsynaptic firing rates,* no synaptic plasticity is induced, i.e. plasticity is "off".

Comment 43

Eqs. 12 to 19: I would like to suggest using the steady state value of ν^I here so that's easier to know how the neuron model's parameters affect this quantity.

Answer: We did not modify Eq 13-20 (old Eq 12-19) because we feel that the current form is more appropriate to convey our results. However, we agree with the reviewer that it is important to point out that ν^I can be replaced by its steady state value. Therefore, we added in line 753: "In Eq 13-20, the inhibitory firing rate can be replaced by its steady state value $\nu^I = N^E \rho^E w^{IE} + \rho^I$." And we explicitly show the equation for the steady state (Eq 6) and the line attractor (Eq 7) in Eq 21 and Eq 22.

Comment 44

Line 634/635 and Eqs. 18 and 19: "The line which separates stable from unstable initial weights can be calculated by taking the ratio of Eq 9 and Eq 10 and equating that to the slope of the line attractor (Eq 5)" Isn't Eq. 5 calculated for the nonlinear inhibitory plasticity model? From Fig 1D: "Black cross marks crossover of the plasticity curves at which weight dynamics become unstable." Isn't the point separating stable from unstable initial weights given by $\dot{w}^{EE} = \dot{w}^{EI}$ for the linear inhibitory plasticity model?

Answer: Since the equation of the line attractor is the same for linear and the nonlinear inhibitory plasticity, the above statement is correct. However, we agree that this might be confusing for the reader. We therefore added in line 751 in the Methods: "*The slope of the line attractor is the same for linear and nonlinear inhibitory plasticity.*"

Comment 45

Eq. 20: I could not find the definition of Δw^{EE} or Δw^{EI}. Is it $\dot{w}^{EE}$ and $\dot{w}^{EI}$? If that's the case, doesn't it make the learning rules non-local? Or would it be that each synapse has a different c post ? I would expect that a sliding threshold defined by the BCM rule, such as in Gjorgjieva et al. (2011), would have similar results, and in this case the learning rule would remain local.

Answer: We thank the reviewer for spotting this inconsistency, we have now updated Eq. 23. We now acknowledge that our dynamic threshold mechanism is non-local, as well as the limitations of our proposal threshold matching mechanism (see also the answers to Comments 4, 6 and 36).

As we already write in response to the other comments, we opted for this alternative implementation of the threshold matching mechanism to prevent it from imposing a target firing rate on the postsynaptic neuron, as is the case with BCM-like rules [4, 14]. Our implementation can then realize a different postsynaptic firing rate depending on the initial conditions of various model parameters, such as the LTD/LTP thresholds (see our response to Comment 23). While we agree that implementing the sliding threshold as in the BCM could solve the non-locality issue of our threshold-matching mechanism, we leave this for future work.

Comment 46

Lines 656 to 664: I find this paragraph a bit hard to follow. First, what results from ref. 26 are the authors linking to their model? Second, it's also not clear to me why using only 100 ms to calculate the points for Fig 5. Third, without any noise all the points can be analytically calculated. Is there a reason not to show the

analytical points?

Answer: First, we now clarify what aspect of ref. 26 can be linked to the model. We write in line 800-801:

> In Fig 5, we link our model to the experimental findings *on how the interaction of excitatory and inhibitory plasticity can lead to fixed E/I ratios [9].*

Second, we choose a time for which not all synaptic weights have reached the line attractor yet (as seen in Fig 5B). For longer times, all weights would reach the line attractor and all points in Fig 5C would be at the gray horizontal line (which corresponds to the analytical E/I weight ratio solution).

Third, indeed the E/I ratios can be calculated at steady state (shown in Fig 5C with the gray horizontal line). However, we wanted to show that depending on the (random) initial condition, the weight change can be very different. As shown in Fig 5C, high E/I before ratios lead to large changes in the E/I after ratios (also reflected in Fig 5E,F). Since D'Amour and Froemke 2015 did a similar analysis in Fig 7 of their manuscript (where undoubtedly the system was not in steady state), we showed the points based on random initial conditions which have not necessarily reached the steady state yet. Therefore, we actually show both, the analytical solution based on the steady state (gray horizontal line in Fig 5C) and the numerics based on random initial conditions where the dynamics have not necessarily reached the steady state (Fig 5B-F).

We now clarify the choice for the chosen time in the Methods in line 809-812:

> *We choose 100 ms so not all synaptic weights have reached the line attractor yet and so we can compare the E/I ratios reached in our model to those measured experimentally [9] which would most likely also not be in steady state.*

Comment 47

Line 668/669: "We define a pattern to mean an increase in the input rate to 4 Hz for 100 ms of four neurons."This sentence is a bit confusing to me. What does mean refer to? Also, how would this be reflected in Eqs. 7 and 8?

Answer: We clarified the term input pattern in lines 835-839:

> *An input pattern is defined by a high firing rate of 4 (Hz) at a subset of four excitatory input neurons for a time of 100 (ms). In Eq 1 and Eq 2, this is reflected by a subset of the N^E inputs having $\rho^E_m = 4$ Hz, where m corresponds to the neurons being part of the respective input pattern. After a time of 100 (ms), a new subset of four excitatory neurons fire at high firing rates.*

Comment 48

Lines 669 to 671: Why 60 seconds? How sensitive are the results to this choice?

Answer: Disinhibition needs to be applied for a sufficiently long time to ensure that inhibitory plasticity can induce competition and form receptive fields. Much longer disihibition will induce no further plasticity. Therefore, the each choice of 60 seconds is arbitrary. We now make this explicit in the text in line 842-844.

> *Disinhibition needs to be applied for a sufficiently long time to ensure that inhibitory plasticity can induce competition and form receptive fields.*

Comment 49

Line 672/673: "... specific inhibitory population as slow and gradual over a time course of 100 s to avoid complete silencing of the postsynaptic excitatory neurons." Would this happen with the linear inhibitory plasticity model as silent postsynaptic activity elicits a decrease in inhibitory weights when inhibitory neurons are active? See point 3.

Answer: Linear inhibitory plasticity can indeed recover the firing rate of the postsynaptic cell by decreasing the inhibitory weight. However, as discussed in Comment 3, this mechanism is not supported by experimental data and choosing a linear inhibitory plasticity rule does not guarantee for stability (as described in Fig 1) and therefore additional mechanisms would be necessary to ensure stability in the context of Fig 7.

Comment 50

Figures: I would like to suggest deleting "in" from "in [Hz]" – isn't it redundant to have "in" and brackets?

Answer: We have removed "in" from the figures.

Comment 51

Figure 4: I could not find the definition of ρ^E_{base} and ρ^E_{distr} in the Methods. Would it be possible to add their definitions in the Methods section?

Answer: Yes, we added a sentence in the Method section in lines 757-759 to define these terms:

> *The perturbations of the presynaptic firing rate ρ^E_{disr} in Fig 4 are defined as instantaneous and permanent increases or decreases from the initial presynaptic firing rate ρ^E_{base}.*

Comment 52

Figure 6 F: It is not clear why the neurons are organised in such a way in this plot. Does the distance between two coupled neurons reflect something?

Answer: In (the new) Fig 7F, the location and distance of individual neurons does not have any meaning. We used the "digraph function" in Matlab which provides an automatic clustering of graphs, which brings similarly tuned neurons closer together in space. Besides making the detection of clusters easier, there is no further meaning in the distance of individual neurons. To make this clear, we added the following sentence in lines 857-859 in the Methods section:

> The clustering graph in Fig 7F (left) was done with the digraph function in Matlab *where the distance between neurons is only used to visualize clusters of neurons with similar tuning.*

Please see also our answer to Comment 11 of Reviewer 1.

Reviewer #3:

Summary

The stability of neural activity requires a certain degree of the robustness of synaptic configuration in neural networks, whereas learning requires flexibility in remodeling the existing synaptic structures. Therefore, stable neural dynamics and learning new experiences have conflicting demands. The authors studied how STDP and homeostatic plasticity interact during the development of neural circuits to organize an excitation-inhibition balance that simultaneously fulfills these demands. Their solution is based on inhibitory plasticity with a

nonlinear learning rule. The authors proposed a class of inhibitory plasticity rules at I-to-E synapses to cancel out excitatory plasticity's inherently positive feedback effects.

Although some results are of potential interest, I have several concerns about the validity of the main results. I cannot exclude the possibility that I have missed some crucial points. However, I feel that the present analysis is not sufficient to confirm the central claim of this study. For the acceptance of this manuscript, the following concerns should be clarified.

Major comments:

Comment 1

The previous linear inhibitory plasticity requires a fine-tuning of the target firing rates of excitatory and inhibitory plasticity rules: $c^E_{post} = c^I_{post}$. In the nonlinear inhibitory plasticity proposed in this manuscript, this condition was relaxed by introducing dynamical modulations of the target firing rates. However, I have difficulty understanding the rules of these modulations. The modulation rules seem to work if $\nu^E > c^E_{post}, c^I_{post}$, and $c^E_{post} < c^I_{post}$ at time $t = 0$. In this case, c^E_{post} is increased, and c^I_{post} is decreased in time; hence $c^E_{post} = c^I_{post}$ is eventually achieved. However, when $\nu^E < c^E_{post}$, $\nu^E < c^I_{post}$, and $c^E_{post} < c^I_{post}, c^E_{post}$ is decreased and c^I_{post} is increased. Therefore, $c^E_{post} = c^I_{post}$ is never reached. Alternatively, when $\nu^E > c^E_{post}, \nu^E < c^I_{post}$, and $c^E_{post} < c^I_{post}$, both c^E_{post} and c^I_{post} are increased. Therefore, whether the desired fine-tuning $c^E_{post} = c^I_{post}$ is achieved is not guaranteed. As the dynamic fine-tuning of the thresholds is central to the proposed nonlinear learning rule, a more rigorous analysis of its properties is necessary.

Answer: We thank the reviewer for their important comment. First, we would like to clarify that Fig 1,2 and 4-7 in the manuscript are with fixed and equal excitatory and inhibitory LTD/LTP thresholds $c^E_{post} = c^I_{post}$. The dynamic threshold matching mechanism, as presented in Fig 3D-F, is one suggestion to avoid the fine tuning of the thresholds when they are assumed to be fixed and identical. We did not want to make this dynamic threshold matching a central point of the manuscript as there may be other implementations of our proposed rule. Nonetheless, we investigated the raised points further.

We now show that for initial conditions other than those in Fig 3D-F the thresholds can also matched and remain stable under perturbations (see S3 Fig). We dissect the concrete example mentioned above: if $\nu^E < c^E_{post}$, $\nu^E < c^I_{post}$, and $c^E_{post} < c^I_{post}$ then it seems that no stability can be achieved because our mechanism would decrease c^E_{post} and increase c^I_{post}, sliding the thresholds in the opposite direction and preventing $c^E_{post} = c^I_{post}$. However, the induction of plasticity also changes the postsynaptic firing rate (see Fig R3). When the postsynaptic rate ν^E is below the thresholds (Fig R3, left), there is both excitatory and inhibitory LTD. Since inhibition is more dominant, this will increase ν^E. Eventually, ν^E will increase above c^E_{post}, changing the dynamics of c^E_{post} (Fig R3, middle). Now there is also excitatory LTP, which increases the postsynaptic rate further. Finally, the postsynaptic rate will also increase above c^I_{post}, also changing the dynamics of c^I_{post} (Fig R3, right). In this case, there is excitatory and inhibitory LTP, which will decrease the postsynaptic rate because inhibition is more dominant than excitation. Now, the dynamics of the two thresholds are on the way to eventually reach $c^E_{post} = c^I_{post}$.

This case can be seen when applying a perturbation which decreases the excitatory input rate (S3 Fig D-F; $\rho^E_{disr} = 1.5$). This first decreases the postsynaptic rate below the excitatory and inhibitory thresholds (S3 Fig D,E) and depresses the excitatory and inhibitory weights (S3 Fig F). The postsynaptic firing rate then increases, which first switches the excitatory threshold dynamics from decreasing to increasing (S3 Fig E), and potentiates the excitatory weights (S3 Fig F). Then the inhibitory threshold dynamics also switches but from increasing to decreasing (S3 Fig E) and potentiates the inhibitory weights (S3 Fig F). Therefore, the firing rates, thresholds and synaptic weights all stabilize (S3 Fig D-F; $\rho^E_{disr} = 1.5$). Similar dynamics but in the opposite direction are seen when applying a perturbation which increases the excitatory input rate (S3 Fig D-F; $\rho^E_{disr} = 2.5$).

We have added a new figure (S3 Fig) and explain the LTD/LTP threshold dynamics in more detail now in the Methods section, lines 768-776:

We point out that modifications in the LTD/LTP thresholds lead to changes in the induction of plasticity as well as the postsynaptic firing rate. For two different initializations of the postsynaptic thresholds, $c^E_{post} < c^I_{post}$ *and* $c^E_{post} > c^I_{post}$*, the weights, rates and postsynaptic threshold dynamics can be stabilized (Fig 3D-F and S3 Fig A-C). The same holds also when applying input perturbations (S3 Fig D-F). For multiple input streams (S3 Fig G-I), the dynamic postsynaptic LTD/LTP thresholds change based on the total excitatory (or inhibitory) weight change, leading to a non-local sliding mechanism which is independent of the input stream.*

Please also see our answer to Comment 2 and Comment 3.

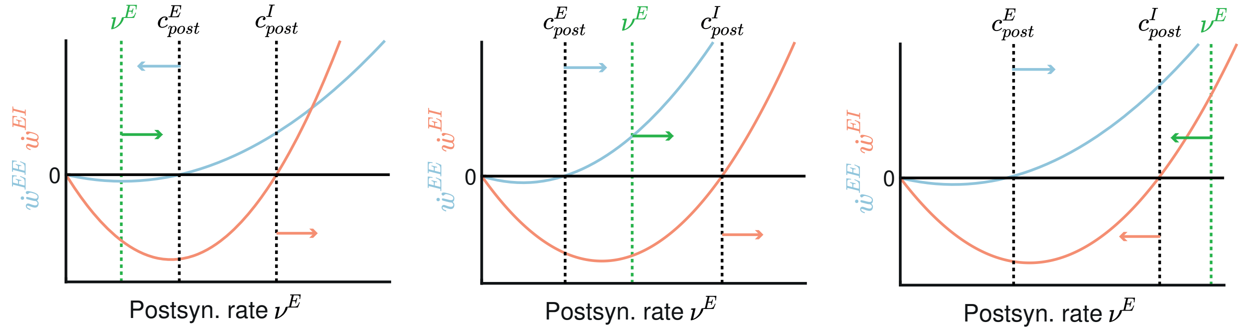

Figure R3: **Schematic of threshold dynamics.** Arrows indicate the dynamics of either the thresholds c^E_{post}, c^I_{post} or the postsynaptic firing rate ν^E. Left: $\nu^E < c^E_{post}$ and $\nu^E < c^I_{post}$, leading to an increase of ν^E, a decrease of c^E_{post} and an increase of c^I_{post}. Middle: $\nu^E > c^E_{post}$ and $\nu^E < c^I_{post}$, leading to an increase of ν^E, an increase of c^E_{post} and an increase of c^I_{post}. Right: $\nu^E > c^E_{post}$ and $\nu^E > c^I_{post}$, leading to a decrease of ν^E, an increase of c^E_{post} and a decrease of c^I_{post}.

Comment 2

Related to the above comment, I wonder whether the initial state should still satisfy the condition $c^I_{post} > c^E_{post}$ even in the dynamical tuning mechanism for c^E_{post} and c^I_{post}. If so, this significantly limits the validity of the proposed non-linear inhibitory plasticity rule. Such cases are not studied in Fig. 3. The tuning procedure should be described mathematically more explicitly in the Methods.

Answer: Indeed, it is not a-priori clear that the starting point $c^I_{post} < c^E_{post}$ also leads to a matching of the thresholds $c^I_{post} = c^E_{post}$. We now explicitly show this in S3 Fig A-C, where $c^I_{post,0} = 0.7$ and $c^E_{post,0} = 1.3$ (flipped case from Fig 3D-F). The thresholds eventually merge (S3 Fig B), while the synaptic weights and the postsynaptic firing rate stabilize (S3 Fig C). A condition for the stabilization is that the weights do not reach their lower bounds at zero, because zero weights prevent plasticity and promote the continuous increase of LTD/LTP thresholds preventing firing rates from stabilizing).

We have added the following text in the Results section, lines 246-249:

The excitatory and inhibitory LTD/LTP thresholds can be matched, and the postsynaptic firing rate and synaptic weights stabilized also for other initializations of the LTD/LTP thresholds (S3 Fig A-C).

Furthermore we have added the following text in the Methods section, lines 770-773:

For two different initializations of the postsynaptic thresholds, $c^E_{post} < c^I_{post}$ *and* $c^E_{post} > c^I_{post}$*, the synaptic weights, postsynaptic firing rate and postsynaptic threshold dynamics can be stabilized (Fig 3D-F and S3 Fig A-C). The same also holds when applying input perturbations (S3 Fig D-F).*

and in the Methods section, lines 776-778:

> *A condition for the stabilization is that the weights do not reach their lower bounds at zero, because zero weights prevent plasticity and promote the continuous increase of LTD/LTP thresholds preventing firing rates from stabilizing).*

Please also see our answer to Reviewer 2 Comment 20 and Reviewer 3 Comment 1 and Comment 3.

Comment 3

For the reasons mentioned in comments 1 and 2, the perturbation analysis in Fig. 4 should be conducted with the dynamical tuning mechanism of the threshold values without assuming the equality $c^I_{post} = c^E_{post}$. These simulations, however, will not be necessary if the authors prove the threshold matching for arbitrary initial values of the variables and parameters.

Answer: Following the reviewer's suggestion, we repeated the perturbation analysis from Fig 4 with dynamic thresholds in our new supplementary figure (S3 Fig D-F). Independent of the direction of the input perturbation, the thresholds converge to the same value (S3 Fig E) leading to stable postsynaptic firing rates (S3 Fig D) and synaptic weights (S3 Fig F).

We have added the following text in lines 291-293:

> *Even when implementing the plasticity rules with dynamic thresholds, performing the perturbations still leads to stable weight and rate configurations (S3 Fig D-F).*

Please also see our answer to Comment 1 and Comment 2. It is difficult to mathematically prove that this holds under all scenarios. Nonetheless, we do not consider the exact threshold matching mechanism as the main result of our paper, rather, the nonlinear inhibitory plasticity rule with its many advantageous properties. The rule assumes that the LTD/LTP thresholds for excitatory and inhibitory plasticity need to be the same, and our proposed matching mechanism is one possibility for how this might be achieved.

Comment 4

In the paragraph on ll. 402-420, the authors discussed the possible relationship between their homeostatic mechanism via inhibitory plasticity rule and the conventional BCM theory, assuming the existence of slow and fast homeostatic mechanisms. The authors suggested that the slow mechanism depends on the cell's intrinsic excitability or synaptic scaling while the fast mechanism on disinhibition and inhibitory plasticity (the cases studied in their model). Please cite references, if any, which give supportive evidence for the different time scales in intrinsic excitability/ synaptic scaling and disinhibition/inhibitory plasticity.

Answer: We now provide the relevant references for the different timescales of synaptic scaling and inhibitory plasticity in the Discussion in line 486-492:

> Slow homeostasis has been linked to *synaptic scaling which we (and others, e.g. [18]) hypothesize to be a possible mechanism behind changes in the postsynaptic threshold. It is usually observed on the timescales of many hours to days [36, 19, 15] but can also occur on the timescale of a few hours [17].* Fast homeostasis might be linked to disinhibition and inhibitory plasticity [13], *which is induced on the timescale of minutes [9, 10, 5].*

Comment 5

The authors vaguely argued the consistency between the computational results and experimental results in several places. For instance, on ll. 441-442, the authors mentioned, "We found that the new nonlinear inhibitory plasticity rule achieves an E/I ratio set point (Fig. 5) in agreement with experimental data [26]. However, in

what sense is the theoretically obtained set point consistent with the experimental observations? Although I do not list all these places, I want to see more precise statements.

Answer: Thanks for this important comment. We have now gone through the manuscript and made the connection between our modeling and experimental results more concrete. Specifically:

Line 318-320 in the Results and also in line 422-423 and 545-546 in the Discussion:

> . . . *in the mouse auditory cortex where inducing excitatory and inhibitory plasticity generates an E/I ratio set-point* [9].

Line 592-594 in the Discussion:

> This is in agreement with various experimental data *indicating that similarly responsive neurons are more strongly connected* [23, 22, 28, 25].

Lines 361-363 in the Results:

> *This shift in the model is in agreement with experimental studies in the hippocampus which have shown that the thresholds between the induction of LTD and LTP are synapse-specific [16, 2].*

Lines 187-191 in the Results (also related to Reviewer 2 Comment 3):

> Differently from the linear inhibitory plasticity rule (Eq 4), the nonlinear inhibitory plasticity rule ensures that I-to-E synapses do not change in the case where the postsynaptic firing rate is zero (Fig 2B, beyond gray line), as *shown in experiments where postsynaptic activity or depolarization is needed to induce inhibitory plasticity* [40].

Minor comments:

Comment 6

In the legend of Fig. 2C, please explain the meanings of solid and dashed lines although we can guess the meanings.

Answer: We have implemented the reviewer's suggestion.

Comment 7

On lines 494-504, the authors suggested that specific and nonspecific inhibitory neurons in the model correspond to SOM+ interneurons and PV+ interneurons, respectively. However, I doubt whether this assumption is biologically plausible from the viewpoint of neuroanatomy. The model's network structure suggests that the specific inhibitory neurons, which target specific excitatory neurons, are the dominant inhibitory neuron type, i.e., PV+ interneuron in local cortical circuits.

Answer: We agree with the reviewer that from the point of connectivity, PVs are considered to target specific excitatory neurons (e.g. [48]). We now acknowledge this alternative interpretation of interneuron type in our model in the discussion in lines 619-622:

> *In contrast to this interpretation, the specific inhibitory neurons in our model might be interpreted as PV neurons. This is supported by experimental evidence which shows that PV neurons strongly inhibit pyramidal neurons which have similar selectivity [48].*

References

[1] Wickliffe C. Abraham. Metaplasticity: Tuning synapses and networks for plasticity. Nature Reviews Neuroscience, 9:387–399, 2008.

[2] Wickliffe C. Abraham and Mark F. Bear. Metaplasticity: plasticity of synaptic plasticity. Trends Neuroscience, 19:126–130, 1996.

[3] Wickliffe C. Abraham and Ann Huggett. Induction and reversal of long-term potentiation by repeated high-frequency stimulation in rat hippocampal slices. Hippocampus, 7:137–145, 1997.

[4] Elie L. Bienenstock, Leon N. Cooper, and Paul W. Munro. Theory for the development of neuron selectivity: orientation specificity and binocular interaction in visual cortex. The Journal of Neuroscience, 2(1):32–48, 1982.

[5] Martha Canto-Bustos, F. Kathryn Friason, Constanza Bassi, and Anne-Marie M. Oswald. Disinhibitory circuitry gates associative synaptic plasticity in olfactory cortex. Journal of Neuroscience, 42(14):2942–2950, 2022.

[6] Renee Chasse, Alexey Malyshev, Roslyn Holly Fitch, and Maxim Volgushev. Altered heterosynaptic plasticity impairs visual discrimination learning in adenosine A1 receptor knock-out mice. Journal of Neuroscience, 41(21):4631–4640, 2021.

[7] Jen-Yung Chen, Peter Lonjers, Christopher Lee, Marina Chistiakova, Maxim Volgushev, and Maxim Bazhenov. Heterosynaptic Plasticity Prevents Runaway Synaptic Dynamics. Journal of Neuroscience, 33(40):15915–15929, 2013.

[8] Leon N. Cooper and Mark F. Bear. The BCM theory of synapse modification at 30: interaction of theory with experiment. Nature Reviews Neuroscience, 13(11):798–810, 2012.

[9] James A. D'amour and Robert C. Froemke. Inhibitory and Excitatory Spike-Timing-Dependent Plasticity in the Auditory Cortex. Neuron, 86:514–528, 2015.

[10] Rachel E. Field, James A. D'amour, Robin Tremblay, Christoph Miehl, Bernardo Rudy, Julijana Gjorgjieva, and Robert C. Froemke. Heterosynaptic Plasticity Determines the Set Point for Cortical Excitatory-Inhibitory Balance. Neuron, 106(5):842–854, 2020.

[11] Robert C. Froemke, Michael M. Merzenich, and Christoph E. Schreiner. A synaptic memory trace for cortical receptive field plasticity. Nature, 450:425–429, 2007.

[12] Stefano Fusi. Computational models of long term plasticity and memory. arXiv, 2017.

[13] Melanie A. Gainey and Daniel E. Feldman. Multiple shared mechanisms for homeostatic plasticity in rodent somatosensory and visual cortex. Philosophical Transactions of the Royal Society B: Biological Sciences, 372(1715), 2017.

[14] Julijana Gjorgjieva, Claudia Clopath, Juliette Audet, and Jean-Pascal Pfister. A triplet spike-timing-dependent plasticity model generalizes the Bienenstock-Cooper-Munro rule to higher-order spatiotemporal correlations. Proceedings of the National Academy of Sciences, 108(48):19383–19388, 2011.

[15] Keith B. Hengen, Mary E. Lambo, Stephen D. VanHooser, Donald B. Katz, and Gina G. Turrigiano. Firing rate homeostasis in visual cortex of freely behaving rodents. Neuron, 80(2):335–342, 2013.

[16] Yan-You Huang, Asuncion Colino, David K. Selig, and Robert C. Malenka. The influence of prior synaptic activity on the induction of long-term potentiation. Science, 255:730–733, 1992.

[17] Keiji Ibata, Qian Sun, and Gina G. Turrigiano. Report Rapid Synaptic Scaling Induced by Changes in Postsynaptic Firing. Neuron, 57:819–826, 2008.

[18] Tara Keck, Mark Hübener, and Tobias Bonhoeffer. Interactions between synaptic homeostatic mechanisms: an attempt to reconcile BCM theory, synaptic scaling, and changing excitation/inhibition balance. Current Opinion in Neurobiology, 43:87–93, 2017.

[19] Tara Keck, Georg B. Keller, R. Irene Jacobsen, Ulf T. Eysel, Tobias Bonhoeffer, and Mark Hübener. Synaptic scaling and homeostatic plasticity in the mouse visual cortex in vivo. Neuron, 80:327–334, 2013.

[20] Jan H. Kirchner and Julijana Gjorgjieva. Emergence of local and global synaptic organization on cortical dendrites. Nature Communications, 12:4005, 2021.

[21] Alfredo Kirkwood, Marc G. Rioult, and Mark F. Bear. Experience-dependent modification of synaptic plasticity in visual cortex. Nature, 381:526–528, 1996.

[22] Ho Ko, Lee Cossell, Chiara Baragli, Jan Antolik, Claudia Clopath, Sonja B. Hofer, and Thomas D. Mrsic-Flogel. The emergence of functional microcircuits in visual cortex. Nature, 496:96–100, 2013.

[23] Ho Ko, Sonja B. Hofer, Bruno Pichler, Katherine A. Buchanan, Per Jesper Sjöström, and Thomas D. Mrsic-Flogel. Functional specificity of local synaptic connections in neocortical networks. Nature, 473:87–91, 2011.

[24] Min Ching Kuo and Hans C. Dringenberg. Short-term (2 to 5 h) dark exposure lowers long-term potentiation (LTP) induction threshold in rat primary visual cortex. Brain Research, 1276:58–66, 2009.

[25] Wei Chung Allen Lee, Vincent Bonin, Michael Reed, Brett J. Graham, Greg Hood, Katie Glattfelder, and R. Clay Reid. Anatomy and function of an excitatory network in the visual cortex. Nature, 532(7599):370–374, 2016.

[26] Ashok Litwin-Kumar and Brent Doiron. Formation and maintenance of neuronal assemblies through synaptic plasticity. Nature Communications, 5(5319), 2014.

[27] Christoph Miehl, Sebastian Onasch, Dylan Festa, and Julijana Gjorgjieva. Formation and computational implications of assemblies in neural circuits. The Journal of Physiology, in press, 2022.

[28] Jae Eun Kang Miller, Inbal Ayzenshtat, Luis Carrillo-Reid, and Rafael Yuste. Visual stimuli recruit intrinsically generated cortical ensembles. Proceedings of the National Academy of Sciences of the United States of America, 111:E4053–E4061, 2014.

[29] Kenneth D. Miller and David J. C. MacKay. The Role of Constraints in Hebbian Learning. Neural Computation, 6:100–126, 1994.

[30] Lisandro Montangie, Christoph Miehl, and Julijana Gjorgjieva. Autonomous emergence of connectivity assemblies via spike triplet interactions. PLoS Computational Biology, 16(5):e1007835, 2020.

[31] Erkki Oja. Simplified neuron model as a principal component analyzer. Journal of Mathematical Biology, 15(3):267–273, 1982.

[32] Benjamin D. Philpot, Juan S. Espinosa, and Mark F. Bear. Evidence for altered NMDA receptor function as a basis for metaplasticity in visual cortex. Journal of Neuroscience, 23(13):5583–5588, 2003.

[33] Sébastien Royer and Denis Paré. Conservation of total synaptic weight through balanced synaptic depression and potentiation. Nature, 422:518–522, 2003.

[34] Auguste Schulz, Christoph Miehl, Michael J. Berry II, and Julijana Gjorgjieva. The generation of cortical novelty responses through inhibitory plasticity. eLife, 10:e65309, 2021.

[35] Taro Toyoizumi, Megumi Kaneko, Michael P. Stryker, and Kenneth D. Miller. Modeling the Dynamic Interaction of Hebbian and Homeostatic Plasticity. Neuron, 84:497–510, 2014.

[36] Gina G. Turrigiano. The Self-Tuning Neuron: Synaptic Scaling of Excitatory Synapses. Cell, 135:422–435, 2008.

[37] Gina G. Turrigiano. The dialectic of hebb and homeostasis. Philosophical Transactions of the Royal Society B: Biological Sciences, 372:20160258, 2017.

[38] Lawrence C Udeigwe, Paul W Munro, and G Bard Ermentrout. Emergent Dynamical Properties of the BCM Learning Rule. The Journal of Mathematical Neuroscience, 7(1), 2017.

[39] Maxim Volgushev, Jen-Yung Chen, Vladimir Ilin, Roman Goz, Marina Chistiakova, and Maxim Bazhenov. Partial Breakdown of Input Specificity of STDP at Individual Synapses Promotes New Learning. The Journal of Neuroscience, 36(34):8842–8855, 2016.

[40] Lang Wang and Arianna Maffei. Inhibitory Plasticity Dictates the Sign of Plasticity at Excitatory Synapses. Journal of Neuroscience, 34(4):1083–1093, 2014.

[41] G. White, W. B. Levy, and O. Steward. Spatial overlap between populations of synapses determines the extent of their associative interaction during the induction of long-term potentiation and depression. Journal of Neurophysiology, 64(4):1186–1198, 1990.

[42] Yue Kris Wu, Keith B. Hengen, Gina G. Turrigiano, and Julijana Gjorgjieva. Homeostatic mechanisms regulate distinct aspects of cortical circuit dynamics. Proceedings of the National Academy of Sciences, 117(39):24514–24525, 2020.

[43] Pierre Yger and Matthieu Gilson. Models of Metaplasticity: A Review of Concepts. Frontiers in Computational Neuroscience, 9(138), 2015.

[44] Friedemann Zenke, Everton J. Agnes, and Wulfram Gerstner. Diverse synaptic plasticity mechanisms orchestrated to form and retrieve memories in spiking neural networks. Nature Communications, 6(6922), 2015.

[45] Friedemann Zenke and Wulfram Gerstner. Hebbian plasticity requires compensatory processes on multiple timescales. Philosophical Transactions of the Royal Society B: Biological Sciences, 372(1715):20160259, 2017.

[46] Friedemann Zenke, Wulfram Gerstner, and Surya Ganguli. The temporal paradox of Hebbian learning and homeostatic plasticity. Current Opinion in Neurobiology, 43:166–176, 2017.

[47] Friedemann Zenke, Guillaume Hennequin, and Wulfram Gerstner. Synaptic Plasticity in Neural Networks Needs Homeostasis with a Fast Rate Detector. PLoS Computational Biology, 9(11):e1003330, 2013.

[48] Petr Znamenskiy, Mean-hwan Kim, Dylan R. Muir, Florencia Iacaruso, Sonja B. Hofer, and Thomas D. Mrsic-Flogel. Functional selectivity and specific connectivity of inhibitory neurons in primary visual cortex. bioRxiv, 294835, 2018.
