# Supplementary material for: Stability and learning in excitatory synapses by nonlinear inhibitory plasticity
Source: PLoS Comput Biol. 2022 Dec 2;18(12):e1010682. doi: 10.1371/journal.pcbi.1010682 (PMC9718420; doi:10.1371/journal.pcbi.1010682)
Supplement: Attachment — Submitted filename: Review2.pdf [file pcbi.1010682.s007.pdf]

The authors have substantially improved the manuscript, and I highly appreciate all the time taken to address my questions. The new simulations with varying pre- and postsynaptic rates complement the analysis of their model and strengthen the manuscript. All my points have been well addressed, however I still have two major and a few minor points to be addressed by the authors. These points are detailed below.

Major points

1. *Varying inputs (related to my previous points 2 and 6).* In the revised version of the manuscript, the authors have shown that when the postsynaptic firing-rate varies over time, both weight and activity are stable (these quantities do not diverge nor go to zero). Given that one of the learning rules has an unstable fixed-point, when the presynaptic activity varies over time (either through noise or oscillatory activity pattern) the weight dynamics is not stable, as shown by new simulations. The authors state that "additional homeostatic mechanisms are necessary to regulate synaptic weight dynamics over longer timescales", yet they presented a possible mechanisms in their manuscript – dynamic c^E_{post} and c^I_{post}. In their response to my point 6, they showed a simulation in which both c^E_{post} and c^I_{post} are dynamic and, as a consequence, the instability is gone. I consider this an important result which I suggest to be included in the manuscript for two reasons. First, these results give an appealing justification for why both c^E_{post} and c^I_{post} would need to change over time, which was lacking in the original submission. Second, it shows that the dynamics that the authors have suggested for c^E_{post} and c^I_{post} are indeed sufficient for stability of weight dynamics in a general condition in which pre- and postsynaptic activities vary over time.

2. *Fixed E/I ratio (related to my previous points 7 and 30).* I appreciate the author's clarification of the concept of fixed E/I ratio. In the resubmitted manuscript, the authors refer to "E/I ratio set-point for fixed input rates", which in my view is not a set-point, but rather a function of input rates. In a more realistic scenario (such as in Fig. 7), the firing-rates are not constant, but change over time according to, e.g., sensory stimuli. If the input rates are not constant (and thus variables in the mathematical sense), the E/I ratio is a function of the variables corresponding to the input rates, $R^{E/I} = f(\rho^E, \rho^I, \ldots)$. In this sense, it would be more accurate to refer to E/I ratio as a *function of input rates*, and not *set-point* because it might be misleading, especially given that the subsection title is "The nonlinear inhibitory plasticity rule establishes an excitatory-to-inhibitory weight ratio set-point". Using the author's definition of E/I ratio set-point (w^{EE}/w^{EI}), any combination of stable excitatory and inhibitory learning rules will establish an E/I ratio set-point, such as the linear inhibitory learning rule together with the unstable excitatory learning rule (Eqs 4 and 3) for certain initial conditions.

   (Related to my previous point 30): Considering the approximation for large w^{EI}, i.e., $w^{EI} \gg \frac{c_{post}}{N^I(N^E \rho^E w^{IE} + \rho^I)}$, the authors state that (line 786) "This inequality is satisfied for the parameters in Fig 5 (Table 1)." However, from Table 1: $c_{post} = 1$; $N^E = N^I = 1$; $\rho^E = 2$; $\rho^I = 0.5$; and $w^{IE} = 0.5$, and thus $\frac{c_{post}}{N^I(N^E \rho^E w^{IE} + \rho^I)} = 2/3$. Is this correct? From Fig 5B, it looks like $1 < w^{EI} < 4$, and it is thus not obvious whether the assumption used for the approximation in Eq. 9 is actually satisfied. It is still not clear to me why this particular assumption was used, because (i) other assumptions could lead to similar results such as strong w^{IE}, large number of excitatory or inhibitory neurons, etc, and (ii) Eq. 8 would be sufficient to explain the phenomenon.

Minor points

3. Introduction and Discussion: The authors give a good overview of the different mechanisms implemented in various modelling studies (and therefore distinct models) to counteract instabilities caused by Hebbian plasticity, e.g., scaling, weight normalization, heterosynaptic plasticity, etc. However, there is little direct comparison to previous models of inhibitory plasticity, which is the main model type being proposed by the authors. Both introduction and discussion section, in my view, could benefit from a brief summary and/or comparison with previous models of inhibitory plasticity, e.g., asymmetric STDP (Haas et al., 2006; Luz and Shamir, 2012; Wilmes and Clopath, 2019), symmetric STDP (Vogels et al., 2011; Agnes et al., 2020), voltage-dependent (Pedrosa and Clopath, 2020), and co-activity-dependent (Agnes and Vogels, 2021).

4. Line 308: "Paradoxically, the E/I ratio set-point decreases as the presynaptic excitatory rate ρ^E increases" Could the authors elaborate on this paradoxical result? Is it paradoxical because the calculation of the E/I ratio does not involve the firing-rates, but only weights?

5. Line 389: “This leads to unbalanced excitatory and inhibitory plasticity due to the random presentation order of the different inputs thereby generating ...” Fig 7 does not show values for inhibitory weights, just excitatory ones. Could the authors elaborate on this aspect of “unbalanced excitatory and inhibitory plasticity”? It is not clear to me what it refers to.

6. Line 421: “A direct consequence of our inhibitory plasticity is the establishment of an E/I weight ratio set-point (Eq 8), ...” This statement might be misleading (see point 2), because the nonlinear inhibitory learning rule does not necessarily establish an E/I weight ratio set-point.

7. Line 544: “We found that the new nonlinear inhibitory plasticity rule achieves an E/I ratio set-point ...” Please see point 2.

8. Line 560: “... the overlap of excitatory and inhibitory LTD/LTP thresholds lead to an E/I weight ratio set-point ...” Please see point 2.

9. Line 567: Typo: $\tilde{5}$.

References

Agnes, E. J., Luppi, A. I., and Vogels, T. P. (2020). Complementary inhibitory weight profiles emerge from plasticity and allow flexible switching of receptive fields. *Journal of Neuroscience*, 40(50):9634–9649.

Agnes, E. J. and Vogels, T. P. (2021). Interacting synapses stabilise both learning and neuronal dynamics in biological networks. *BioRxiv*.

Haas, J. S., Nowotny, T., and Abarbanel, H. D. I. (2006). Spike-timing-dependent plasticity of inhibitory synapses in the entorhinal cortex. *Journal of Neurophysiology*, 96(6):3305–3313.

Luz, Y. and Shamir, M. (2012). Balancing feed-forward excitation and inhibition via hebbian inhibitory synaptic plasticity. *PLoS Computational Biology*, 8(1):e1002334.

Pedrosa, V. and Clopath, C. (2020). Voltage-based inhibitory synaptic plasticity: network regulation, diversity, and flexibility. *bioRxiv*.

Vogels, T. P., Sprekeler, H., Zenke, F., Clopath, C., and Gerstner, W. (2011). Inhibitory plasticity balances excitation and inhibition in sensory pathways and memory networks. *Science*, 334(6062):1569–1573.

Wilmes, K. A. and Clopath, C. (2019). Inhibitory microcircuits for top-down plasticity of sensory representations. *Nature Communications*, 10(1):1–10.
