# Supplementary material for: Stability and learning in excitatory synapses by nonlinear inhibitory plasticity
Source: PLoS Comput Biol. 2022 Dec 2;18(12):e1010682. doi: 10.1371/journal.pcbi.1010682 (PMC9718420; doi:10.1371/journal.pcbi.1010682)
Supplement: Attachment — Submitted filename: rev_ROUND2.pdf [file pcbi.1010682.s008.pdf]

We thank the reviewer for these additional points. Below we answer each of them and make the corresponding changes in the manuscript in blue. We hope that the reviewer finds the paper now suitable for publication.

Reviewer #2:

Major comments:

Comment 1

Varying inputs (related to my previous points 2 and 6). In the revised version of the manuscript, the authors have shown that when the postsynaptic firing-rate varies over time, both weight and activity are stable (these quantities do not diverge nor go to zero). Given that one of the learning rules has an unstable fixed-point, when the presynaptic activity varies over time (either through noise or oscillatory activity pattern) the weight dynamics is not stable, as shown by new simulations. The authors state that "additional homeostatic mechanisms are necessary to regulate synaptic weight dynamics over longer timescales", yet they presented a possible mechanisms in their manuscript – dynamic c^E_{post} and c^I_{post}. In their response to my point 6, they showed a simulation in which both c^E_{post} and c^I_{post} are dynamic and, as a consequence, the instability is gone. I consider this an important result which I suggest to be included in the manuscript for two reasons. First, these results give an appealing justification for why both c^E_{post} and c^I_{post} would need to change over time, which was lacking in the original submission. Second, it shows that the dynamics that the authors have suggested for c^E_{post} and c^I_{post} are indeed sufficient for stability of weight dynamics in a general condition in which pre- and postsynaptic activities vary over time.

Answer: We believe that there is a misunderstanding in the interpretation of our Figure R2 in response to point 6 in the previous revision. This figure showed that when the postsynaptic firing rate varies over time, using dynamic thresholds c^E_{post} and c^I_{post} still generates stable weight, rate and threshold dynamics (Figure 6A,B). However, when the presynaptic firing rate varies over time, this is not the case and the weights slowly drift (as we show in Figure 6D,F). As we wrote in the corresponding Results section, the difference between the two scenarios can be seen in the phase planes. In the first case, when the postsynaptic firing rate varies, the line attractor intersects the abscissa but does not change the slope. The weights oscillate between two parallel line attractors and remain bounded. In the second case, when the presynaptic firing rate varies, the line attractor changes the slope. Therefore, the weights oscillate between two line attractors with different slopes and gradually increase. We did some initial explorations to see if any parameter combinations can solve the problem. But short of proposing a completely new mechanism for the dynamic threshold changes, we leave this for future work. As we write in the manuscript, on shorter timescales the fluctuations should not pose a problem since they are small and gradually increase the weights.

Nonetheless, we added Figure R2 from the first revision to the main manuscript (S4 Fig). We discuss the new supplementary figure in the section *Performance of the nonlinear inhibitory plasticity rule under varying presynaptic input and postsynaptic firing rate.*

Comment 2

Fixed E/I ratio (related to my previous points 7 and 30). I appreciate the author's clarification of the concept of fixed E/I ratio. In the resubmitted manuscript, the authors refer to "E/I ratio set-point for fixed input rates", which in my view is not a set-point, but rather a function of input rates. In a more realistic scenario (such as in Fig. 7), the firing-rates are not constant, but change over time according to, e.g., sensory stimuli. If the input rates are not constant (and thus variables in the mathematical sense), the E/I ratio is a function of the variables corresponding to the input rates, $R^{E/I} = f(\rho^E, \rho^I, ...)$. In this sense, it would be more accurate to refer to E/I ratio as a function of input rates, and not set-point because it might be misleading, especially given that the subsection title is "The nonlinear inhibitory plasticity rule establishes an excitatory-to-inhibitory weight ratio set-point". Using the author's definition of E/I ratio set-point (w^{EE}/w^{EI}), any combination of stable excitatory and inhibitory learning rules will establish an E/I ratio set-point, such as the linear inhibitory

learning rule together with the unstable excitatory learning rule (Eqs 4 and 3) for certain initial conditions.

Answer: We now removed the misleading term "set-point" from the manuscript and replaced it at multiple locations with the more accurate term: "fixed E/I weight ratio for constant input rates". Note we already used fixed E/I weight ratio in the first version of the paper but we now add "for constant input rates." We also write that the novel inhibitory plasticity model **affects** E/I weight ratios in the Abstract, without specifying if the ratio is fixed or not.

(Related to my previous point 30): Considering the approximation for large w^{EI}, i.e., $w^{EI} \gg \frac{c_{post}}{N^I(N^E \rho^E w^{IE} + \rho^I)}$, the authors state that (line 786) "This inequality is satisfied for the parameters in Fig 5 (Table 1)." However, from Table 1: $c_{post} = 1; N^E = N^I = 1; \rho^E = 2; \rho^I = 0.5$; and $w^{IE} = 0.5$, and thus $\frac{c_{post}}{N^I(N^E \rho^E w^{IE} + \rho^I)} = 2/3$. Is this correct? From Fig 5B, it looks like $1 < w^{EI} < 4$, and it is thus not obvious whether the assumption used for the approximation in Eq. 9 is actually satisfied. It is still not clear to me why this particular assumption was used, because (i) other assumptions could lead to similar results such as strong w^{IE} , large number of excitatory or inhibitory neurons, etc, and (ii) Eq. 8 would be sufficient to explain the phenomenon.

Answer: We thank the reviewer for this comment. The reviewer is correct, the assumption is not always met. E.g. for very small w^{EI}, $R^{E/I}_\infty$ is not a good approximation for $R^{E/I}$. This can be seen in the inset of Fig. 5A: for small w^{EI} the difference between $R^{E/I}$ and the dashed line (which is $R^{E/I}_\infty$) can be very large. We use the approximation $R^{E/I}_\infty$ to show mathematically that the system reaches a fixed E/I ratio (for constant input rates), because $R^{E/I}_\infty$ is independent of the synaptic weights (in contrast to $R^{E/I}$). But the argument that the system reaches a fixed E/I ratio (for constant input rates) is only true if the synaptic weights after plasticity settle at a sufficiently high value.

A different choice of parameters (as suggested by the reviewer) could minimize the "error" between $R^{E/I}$ and $R^{E/I}_\infty$. We did not explore this further in context of Fig 5 aiming to keep the same parameters as in other figures in our manuscript.

We modified line 310-312 to explain the reason for showing $R^{E/I}_\infty$ and we updated the sentence in the Methods in line 808 to clarify that this condition is only met for sufficiently large inhibitory synaptic weights w^{EI}.

Minor comments:

Comment 3

Introduction and Discussion: The authors give a good overview of the different mechanisms implemented in various modelling studies (and therefore distinct models) to counteract instabilities caused by Hebbian plasticity, e.g., scaling, weight normalization, heterosynaptic plasticity, etc. However, there is little direct comparison to previous models of inhibitory plasticity, which is the main model type being proposed by the authors. Both introduction and discussion section, in my view, could benefit from a brief summary and/or comparison with previous models of inhibitory plasticity, e.g., asymmetric STDP (Haas et al., 2006; Luz and Shamir, 2012; Wilmes and Clopath, 2019), symmetric STDP (Vogels et al., 2011; Agnes et al., 2020), voltage-dependent (Pedrosa and Clopath, 2020), and co-activity-dependent (Agnes and Vogels, 2021).

We now included an in-depth discussion of inhibitory plasticity mechanisms in the Discussion section (line 466) and added Agnes and Vogels, 2021 to line 678. Furthermore we mention some of the computational studies on inhibitory plasticity now in the Introduction at line 59.

Comment 4

Line 308: "Paradoxically, the E/I ratio set-point decreases as the presynaptic excitatory rate ρ^E increases" Could the authors elaborate on this paradoxical result? Is it paradoxical because the calculation of the E/I ratio does not involve the firing-rates, but only weights?

Answer: It is paradoxical in the sense that an increase in excitatory input, by increasing the presynaptic excitatory rate ρ^E, should (naively) lead also to an increase in the E/I ratio. However, here this is not the case, since an increase in ρ^E actually leads to a decrease in the E/I ratio (as can be seen in Eq. 8). But reconsidering this sentence we believe that the word "paradoxiacally" doesn't add anything to the clarity of the manuscript, hence we removed the word from respective sentence.

Comment 5

Line 389: "This leads to unbalanced excitatory and inhibitory plasticity due to the random presentation order of the different inputs thereby generating ..." Fig 7 does not show values for inhibitory weights, just excitatory ones. Could the authors elaborate on this aspect of "unbalanced excitatory and inhibitory plasticity"? It is not clear to me what it refers to.

Answer: We agree with the reviewer, this sentence was a bit confusing. We now rewrote it.

Comment 6

Line 421: "A direct consequence of our inhibitory plasticity is the establishment of an E/I weight ratio set-point (Eq 8), ..." This statement might be misleading (see point 2), because the nonlinear inhibitory learning rule does not necessarily establish an E/I weight ratio set-point.

Answer: We now write more accurately: "...establishment of a fixed E/I weight ratio set-point for constant input rates...".

Comment 7

ine 544: "We found that the new nonlinear inhibitory plasticity rule achieves an E/I ratio set-point ..." Please see point 2.

Answer: We removed the term set-point.

Comment 8

Line 560: "... the overlap of excitatory and inhibitory LTD/LTP thresholds lead to an E/I weight ratio set-point ..." Please see point 2.

Answer: We removed the term set-point.

Comment 9

Line 567: Typo: 5

Answer: We fixed the typo.
